# Selective area doping for Mott neuromorphic electronics

**DOI:** 10.1126/sciadv.ade4838

**Published:** 2023-03-17

**Authors:** Sunbin Deng, Haoming Yu, Tae Joon Park, A. N. M. Nafiul Islam, Sukriti Manna, Alexandre Pofelski, Qi Wang, Yimei Zhu, Subramanian K. R. S. Sankaranarayanan, Abhronil Sengupta, Shriram Ramanathan

**Affiliations:** ^1^ School of Materials Engineering, Purdue University, West Lafayette, IN 47907, USA.; ^2^ School of Electrical Engineering and Computer Science, The Pennsylvania State University, University Park, PA 16802, USA.; ^3^ Center for Nanoscale Materials, Argonne National Laboratory, Lemont, IL 60439, USA.; ^4^ Department of Mechanical and Industrial Engineering, University of Illinois, Chicago, IL 60607, USA.; ^5^ Department of Condensed Matter Physics and Materials Science, Brookhaven National Laboratory, Upton, NY 11973, USA.

## Abstract

The cointegration of artificial neuronal and synaptic devices with homotypic materials and structures can greatly simplify the fabrication of neuromorphic hardware. We demonstrate experimental realization of vanadium dioxide (VO_2_) artificial neurons and synapses on the same substrate through selective area carrier doping. By locally configuring pairs of catalytic and inert electrodes that enable nanoscale control over carrier density, volatility or nonvolatility can be appropriately assigned to each two-terminal Mott memory device per lithographic design, and both neuron- and synapse-like devices are successfully integrated on a single chip. Feedforward excitation and inhibition neural motifs are demonstrated at hardware level, followed by simulation of network-level handwritten digit and fashion product recognition tasks with experimental characteristics. Spatially selective electron doping opens up previously unidentified avenues for integration of emerging correlated semiconductors in electronic device technologies.

## Teaser

Neuromorphic hardware by selective area doping of vanadium dioxide is demonstrated.

## INTRODUCTION

Neuromorphic computing represents a computational paradigm that aims to emulate characteristics of information processing in the brain. Mott semiconductors offer a powerful platform to realize neuromorphic hardware via electrically driven conductance transitions (*1*–*3*). For instance, a single vanadium dioxide (VO_2_) device connected to a capacitor can serve as an oscillatory artificial neuron in a more compact manner (*4*–*7*) compared to traditional silicon (Si) complementary metal-oxide semiconductor layouts. While individual artificial neurons and synaptic primitives have been demonstrated with correlated electron semiconductors demonstrating substantial potential (*8*–*13*), interconnected networks made of neurons and synapses at hardware level and relevant fabrication processes for Mott circuits are in their infancy. While implementation of hardware-based neuromorphic test structures have been reported in literature (*14*–*16*), the interconnected neuronal and synaptic components usually adopted heterotypic material systems and device structures.

We hypothesize that the ability to perform multiple neural functions with the same material system can simplify the chip-scale fabrication process among other benefits. Especially when new materials that have never been introduced in a fab are being considered, if they can offer more than one compelling device application within the same technology domain, then the chances of their consideration naturally become greater. However, this requires innovation in the implementation of diverse neural functions into identical devices after fabrication. For this purpose, we borrow the concept of selective doping from integrated circuit industry and apply them to quantum materials. Selective area doping, which is effective not only for traditional semiconductors (e.g., Si and III-V compounds) but also for emerging materials [e.g., two-dimensional materials (*17*–*19*)], is used across the spectrum of semiconductor technologies to create solid-state devices, such as junctions, contacts, transistors, and inversion layers. Adapting this approach to Mott semiconductors represents a promising direction.

At the outset, we note that there are profound differences in doping physics between band semiconductors such as Si and Mott semiconductors such as VO_2_ (*20*). In Si and related materials, aliovalent doping concentration in the order of parts per million is sufficient for repositioning the Fermi level or band edges for function, whereas in Mott materials, doping density in the order of 0.1 to 1 electron per unit cell (i.e., ~10^5^ to 10^6^ larger doping density) is needed to induce conductance changes (*21*, *22*). Hence, strategies for doping Mott semiconductors that can be scalable and viable for semiconductor technologies are a nontrivial problem.

**Fig. 1.**
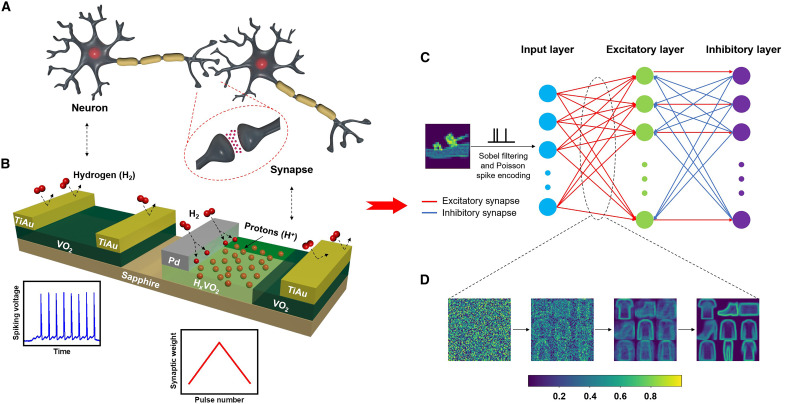
Homotypic Mott neuromorphic platform. (**A**) Schematic of biological neurons and synapses. Neurons serve as processing units connected through synapses. (**B**) Schematic of two-terminal volatile and nonvolatile (H*_x_*)VO_2_ devices integrated on a single sapphire substrate. The volatilities in the (H*_x_*)VO_2_ material system can be controlled by selective hydrogenation. Pristine vanadium dioxide (VO_2_) devices can be used as spiking neurons, and nonvolatile H*_x_*VO_2_ devices can serve as analog synaptic components. (**C**) Network architecture for the (H*_x_*)VO_2_ spiking neural network (SNN) proposed in this work. An example input is from the Fashion-MNIST (Modified National Institute of Standards and Technology) dataset, and it goes through Sobel filtering and Poisson spike encoding before being fed into the network. For MNIST, only the spike encoding is performed before being input to the network. The input layer size is 784, followed by 400 excitatory and inhibitory neurons (1600 for MNIST). (**D**) Weight evolution during training on Fashion-MNIST of nine representative neurons of the network. The weights are modulated according to the spike timing–dependent plasticity (STDP) rule and approximate different patterns in the training set.

The principal objective of this study is to experimentally demonstrate the cointegration of artificial neuronal and synaptic components with homotypic material systems and device structures on chip by spatially selective hydrogen donor doping of VO_2_. This is demonstrated by careful design of catalytic and inert electrode pairs and experimental measurements of electrical properties. Hydrogen as a dopant (*23*–*25*) performs two important and unique functions here: reduces the insulating ground-state resistance and controls the memory state retention (i.e., volatility versus nonvolatility) in two-terminal Mott devices. Hydrogen is selectively doped using catalytic metal electrodes (e.g., Pd), such that the hydrogenated devices can serve as synapses for learning and memory, while pristine undoped devices can function as neurons for signal transfer. Therefore, by spatially selective doping, we are able to realize the two most sought-after neuromorphic functions essential for neural networks with the same parent material. The gentle forming gas (5% H_2_) anneal process causes no lattice damage, and both ground-state resistance and volatility of the memory are tunable. By suitable lithographic design of catalytic and inert electrodes, both volatile and nonvolatile devices with homotypic materials and structures can be integrated on a single chip (Fig. 1, A and B). As proof of principle, neural circuit motifs are then fabricated and demonstrated at hardware level to emulate excitation/inhibition functions. With the experimental characteristics, network-level recognition tasks are further simulated on the Modified National Institute of Standards and Technology (MNIST) (*26*) and Fashion-MNIST (*27*) datasets with test accuracies comparable to other state-of-the-art unsupervised approaches (Fig. 1, C and D).

## RESULTS

### Spatially selective hydrogenation of VO_2_

**Fig. 2.**
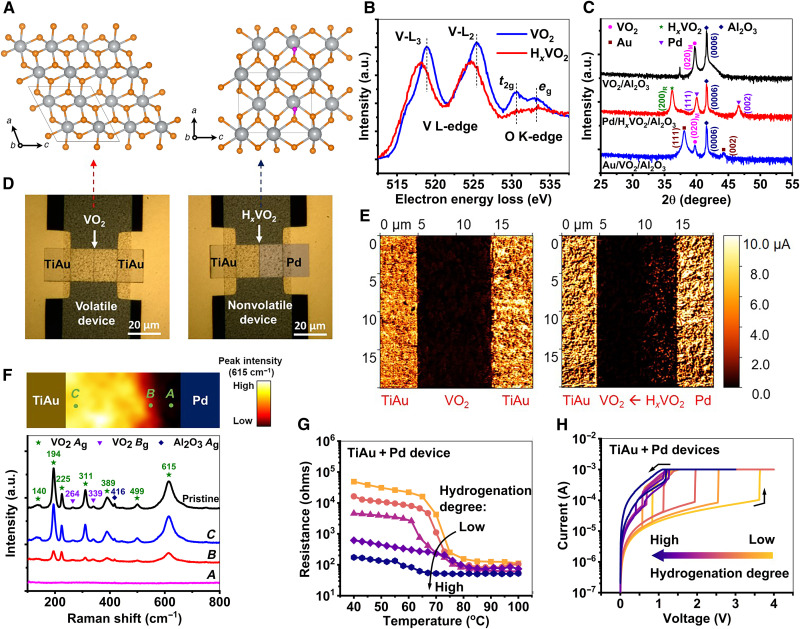
Selective area carrier doping. (**A**) Crystal structures of VO_2_ and H*_x_*VO_2_. Gray, orange, and purple spheres stand for vanadium, oxygen, and hydrogen atoms, respectively. The pristine VO_2_ has a monoclinic structure at room temperature. In H*_x_*VO_2_, hydrogen atoms tend to occupy interstitial sites among VO_2_ lattices. Density functional calculations show that H*_x_*VO_2_ stays in a pseudorutile phase when the hydrogenation level (*x*) is >0.25. (**B**) Averaged electron energy-loss spectroscopy spectra from a VO_2_ and a H*_x_*VO_2_ region centered on the vanadium (V) L_2,3_ and oxygen (O) K-edges. The spectra were normalized using the averaged intensity level between 640 and 660 eV (see fig. S3). a.u., arbitrary units. (**C**) X-ray diffraction (XRD) spectra of the VO_2_ thin film (black line) grown on c-plane sapphire substrate, the H*_x_*VO_2_ with Pd electrodes (red line), and VO_2_ with TiAu electrodes (blue line) after the hydrogenation. The out-of-plane lattice of VO_2_ after the hydrogenation is expanded. (**D**) Optical microscope image of pristine and H-doped VO_2_ devices was fabricated on a single substrate. The channel region is noted with a down pointing arrow. (**E**) Conductive atomic force microscope (C-AFM) images of the (H*_x_*)VO_2_ channels with different electrode combinations after the hydrogenation. Bright conductivity contrast appears near the Pd electrode of TiAu + Pd device, indicating the hydrogenated VO_2_. (**F**) Spatial Raman mapping in the (H*_x_*)VO_2_ channel of TiAu + Pd device (top panel). The Raman peak of VO_2_ at 615 cm^−1^ was fixed for this mapping. This characteristic peak exists in VO_2_ but disappears in H*_x_*VO_2_. Raman spectra measured at points *A*, *B*, and *C* (bottom panel). The Raman spectrum of monoclinic VO_2_ is plotted as a reference. (**G**) Temperature-dependent resistance of the TiAu + Pd device was annealed at different annealing conditions. (**H**) Current-voltage relation (*I*-*V*) curves of the TiAu + Pd device for different hydrogenation levels. Hydrogenation can effectively create intermediate resistance states in VO_2_.

VO_2_ can be hydrogen-doped via a variety of methods, such as high-temperature annealing (*28*), electrochemical insertion (*10*, *29*), and catalytic spillover (*23*, *24*). This work uses the catalytic spillover method to demonstrate selective doping, which occurs at back-end compatible low process temperatures (fig. S1). Palladium (Pd) is a commonly used catalytic electrode in hydrogen spillover process (*30*), and it is also effective toward catalytic hydrogen doping of VO_2_ (*31*, *32*). The inserted hydrogen dopants (i.e., protons) occupy interstitial sites among VO_2_ lattices and bond with oxygen atoms along with electron transfer (Fig. 2A and fig. S2). The electron doping effect can be observed in the vanadium L_2,3_ and oxygen K-edges from the electron energy loss spectra in Fig. 2B and fig. S3. The relative intensity ratio of *t*_2g_ peak over *e*_g_ peak in hydrogenated VO_2_ (H*_x_*VO_2_) is much decreased compared with that in pristine VO_2_, indicating that more states at the lower *t*_2g_ level are filled by electrons. Meanwhile, the vanadium L_2,3_ edge shows a red shift, which is consistent with the valence state change from V^4+^ to V^3+^ as a result of higher V 3d orbital occupancy (*33*). X-ray diffraction (XRD) spectra (Fig. 2C) also reflect the Pd catalyst–assisted hydrogen spillover process. A 50-nm-thick pristine VO_2_ thin film has a monoclinic (020)_M_ peak at 39.8° [equivalent to a rutile (200)_R_ peak] at room temperature. After the hydrogenation with Pd electrodes, the diffraction peak of (200)_R_ shifts to 36.2°, indicating the out-of-plane lattice expansion (*23*).

Compared with the Pd electrode, the TiAu electrode is less active in the hydrogen spillover process (*34*). The VO_2_ film with the TiAu electrodes remains identical in its XRD spectrum after the hydrogenation, and the region near TiAu is still in its original gray color (indexed as VO_2_) (fig. S4). It indicates that negligible doping occurs at the TiAu-VO_2_-H_2_ triple-phase boundary. Therefore, by selecting the combination of catalytic (Pd) and inert (TiAu) electrodes, proton distribution across the device channels in two-terminal VO_2_ devices (Fig. 2D) can be controlled. To visualize the proton distribution, the surface current of devices with different electrode pairs were probed using a conductive atomic force microscope (C-AFM) after the hydrogenation (fig. S5). For the VO_2_ device with two inert TiAu electrodes (i.e., TiAu + TiAu device), the probed current in the channel shows low intensity, indicating an insulating nature of VO_2_ and that no protonation occurred during the hydrogenation. In contrast, for the VO_2_ device with Pd electrodes (i.e., Pd + Pd device), the probed current becomes higher, indicating proton distribution across the whole channel. The Pd + Pd device shows metallic H*_x_*VO_2_ behavior, where both thermally and electrothermally driven insulator-metal transition phenomena are not observed (figs. S6 and S7). Here, we refer to electrothermally driven insulator-metal transition as E-IMT. For the device with an asymmetric electrode pair (i.e., TiAu + Pd device), the probed current decreases monotonically across the channel from Pd to TiAu electrodes, as shown in Fig. 2E. The proton distribution was further mapped using Raman spectroscopy. All the peaks observed in the channel are in good agreement with the reported monoclinic VO_2_ results (*35*). On the other hand, the metallic rutile H*_x_*VO_2_ has no Raman peaks in this range (100 to 800 cm^−1^) (*36*). Thus, by comparing the intensity of characteristic VO_2_ peak (at 615 cm^−1^) measured at different locations within the channel, a decrease of proton density across the channel from Pd to TiAu was observed, indicating a transition from H*_x_*VO_2_ to VO_2_ (Fig. 2F and fig. S8). By subtly controlling hydrogenation level and proton distribution, more intermediate states are accessible. Figure 2G shows temperature-dependent resistance of the device with asymmetric electrode pairs at different hydrogenation levels. The ground-state resistance continues to decrease with increase of hydrogenation time and eventually becomes comparable to the resistance after the phase transition. The hydrogenation also tunes the electrothermally driven transition behavior of H*_x_*VO_2_ device (Fig. 2H and fig. S9). Depending on the hydrogen doping, threshold voltage (*V*_th_) for triggering the E-IMT decreases from ~3.6 to ~0.6 V. Further hydrogenation can induce a nonvolatile current-voltage relation (*I*-*V*) hysteresis characteristic of the device. Such a nonvolatility is reversible, and the volatile E-IMT phenomenon is reproducible in dehydrogenated devices (fig. S10).

### Volatile and nonvolatile memory in (H*_x_*)VO_2_ devices

**Fig. 3.**
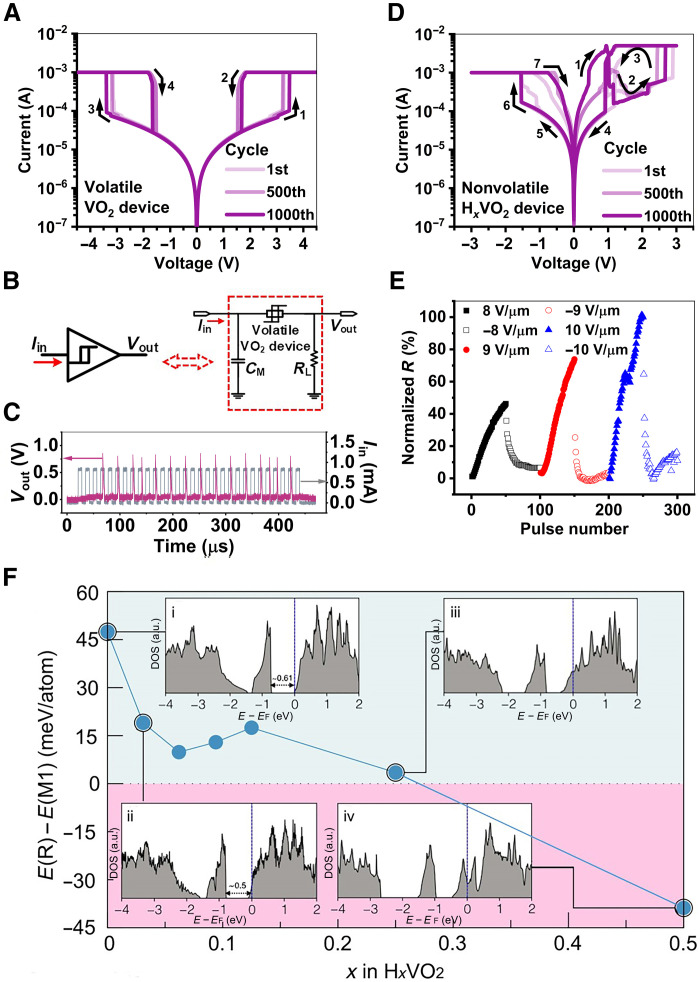
Threshold switching and learning in (H*_x_*)VO_2_ devices. (**A**) *I*-*V* curves of the TiAu + TiAu device in the 1st, 500th, and 1000th sweep cycle with a compliance current (*I*_cc_) of 1 mA. The TiAu + TiAu device clearly demonstrates volatile threshold switching. (**B**) Schematic of neuronal component composed of the volatile VO_2_ device, a load resistor, and a capacitor. (**C**) Measured output voltage waveforms of the neuron. Amplitutde (*A*_p_), capacitor (*C*_M_), pulse width (*t*_p_), and duty ratio (*D*_p_) are set at 0.8 mA, 4.4 nF, 7 μs, and 50%, respectively. (**D**) *I*-*V* curves of the TiAu + Pd device in the 1st, 500th, and 1000th sweep cycle with an *I*_cc_ of 5 mA in the positive polarity and 1 mA in the negative polarity. The TiAu + Pd device exhibits a combination of nonvolatile resistive switching and threshold switching. (**E**) Potentiation and depression of the TiAu + Pd device upon different applied pulses. The pulse widths are 1 μs. (**F**) First-principles calculation of relative stabilities of H*_x_*VO_2_ (M1) and H*_x_*VO_2_ (R) phases across different levels of H concentrations. For the sake of clarity, H*_x_*VO_2_ (M1) and H*_x_*VO_2_ (R) are in pseudomonoclinic and pseudorutile phases, respectively, since the presence of protons alters the ideal lattice parameters and bond distances of both the pristine monoclinic and rutile phases. The light sea green region describes the zone where H*_x_*VO_2_ (M1) is more favorable, whereas the light magenta regime indicates that H*_x_*VO_2_ (R) is more stable. Insets (i), (ii), (iii), and (iv) are the total density of states (DOS) of the low-energy phases of 0.0, 0.03, 0.25, and 0.50 atomic fraction of H levels in H*_x_*VO_2_, respectively. With increasing H dopant level, the semiconducting monoclinic H*_x_*VO_2_ phase (i) narrows its bandgap from ~0.61 to 0.5 eV as seen in (ii). Upon further doping, it changes to metallic (iii) in its pseudomonoclinic phases and its pseudorutile phases (iv).

With different combinations of Pd and TiAu electrodes, the VO_2_ devices can show tunable volatility enabled by different proton distributions across the channels after the selective hydrogenation. The TiAu + TiAu devices retain the pristine VO_2_ channels and exhibit threshold switching with symmetric *I*-*V* hysteresis loops in both positive and negative bias polarities, as shown in Fig. 3A and fig. S11. *V*_th_, above which the E-IMT is triggered by Joule heating due to the flowing current, and holding voltage (*V*_hold_), below which the relaxation happens, are ±3.2 and ±1.5 V on average, respectively. The devices retain a clear gap between *V*_th_ and *V*_hold_ for reliable operation (figs. S12 and S13). The devices show stable volatile characteristics with no resistance changes after multicycle electric pulse stimuli are applied (Fig. S14). These results indicate that the TiAu + TiAu device can be used to emulate artificial neurons, and the well-known leaky integrate and fire (LIF) neuron functions are demonstrated, as shown in Fig. 3B. The TiAu + TiAu device is connected to a load resistor (*R*_L_) in series and a capacitor (*C*_M_) in parallel. Current pulses are applied to the circuit and once the potential across the TiAu + TiAu device reaches *V*_th_ through *C*_M_ charging, the device can be switched from the off-state to the on-state with a sudden increase of current flow (Fig. 3C). The firing rate is tunable upon the capacitor and the input current pulses [including pulse width (*t*_p_), amplitude (*A*_p_), and duty ratio (*D*_p_)] (figs. S15 to S19). Apart from current pulses, voltage pulses are also effective to activate the LIF neuron circuit and to modulate the firing behavior (fig. S20).

On the other hand, the TiAu + Pd devices show a nonvolatile characteristic after the hydrogenation. As shown in Fig. 3D, there is a combination of nonvolatile resistive switching and volatile threshold switching (with a *V*_th_ of ~2.4 V) in the positive bias polarity of *I*-*V* curves even after 1000 sweeps. Considering that the undoped TiAu + Pd devices exhibit threshold switching only, this unique phenomenon should be attributed to the partially hydrogen-doped channels, which are a mixture of VO_2_ and H*_x_*VO_2_. Because protons are able to drift in the channel under an external electric field, we can observe the changes of device resistance upon consecutive *I*-*V* sweeps and multicycle electric pulses, and the devices maintain their resistance states for >10^4^ s after the removal of electrical stimuli (figs. S21 to S23). Such a nonvolatile feature widely exists in the doped TiAu + Pd devices with reasonable cycle-to-cycle and device-to-device variations (fig. S24).

The insertion of protons into the H*_x_*VO_2_ channels introduces intermediate resistance states to the hydrogenated TiAu + Pd devices, which can be used to design artificial synapses. As shown in fig. S25A, upon consecutive positive or negative electric pulse stimuli, device resistance and hence synaptic weight (*w*) can be repeatedly programmed. The change of device resistance is dependent on the applied voltage pulse amplitude and width. For example, when the pulse amplitude increases from 8 to 10 V/μm, the updated resistance after 50 consecutive pulses is doubled (Fig. 3E). When the pulse width increases from 1 to 10 μs, the resistance difference after 50 consecutive pulses reaches about nine times (fig. S25B). Under different pulse conditions, the TiAu + Pd devices perform a relatively linear resistance potentiation with an extracted nonlinear factor of ~0.8 (fig. S26). This is beneficial for the enhancement of training accuracy in spiking neural networks (SNNs) using the hydrogenated TiAu + Pd devices as synapses (*37*).

The multiple metastable states in the devices and their impact on the resistance states are elucidated by density functional first-principles calculations. Figure 3F shows the relative stability analysis based on the total energy differences between pseudomonoclinic H*_x_*VO_2_ (M1) and pseudorutile H*_x_*VO_2_ (R) phases with different concentrations of H dopants. The simulation results reveal that the pseudomonoclinic phase (light sea green region) is more favorable when *x* is ≤0.25 atomic fraction of a H*_x_*VO_2_ formula unit. Upon further doping, the pseudorutile phase becomes more stable (light magenta regime). By accessing the electronic properties in terms of the total and partial density of states (DOS) at different hydrogenation levels, we observe that the intermediately doped H*_x_*VO_2_ (R) phase retains a metallic characteristic with increasing H doping (0.25 < *x* ≤ 0.5), while the increase of H content in the lightly doped H*_x_*VO_2_ (M1) phase reduces its bandgap before transitioning into this metallic state (fig. S27). The reduction of bandgap is attributed to the partial occupation of *d*_||_*/π* orbitals upon the addition of hydrogen and is reflected in the shifting down of the occupancy of *d*_||_ (*d*_*x*2−*y*2_) orbitals (fig. S28). Across the lightly hydrogenated TiAu + Pd device channels, there should be a portion of the semiconducting H*_x_*VO_2_ region with *x* value ranging between 0 and 0.25. Upon application of external electric field, the migration of protons results in the update of the *x* value in H*_x_*VO_2_ locally. Because the change of bandgap affects the resistivity of the semiconducting regions, their redistribution can bring about tunable intermediate resistance states to the synaptic devices.

### Design and fabrication of cointegrated neural circuit motifs with (H*_x_*)VO_2_ devices

**Fig. 4.**
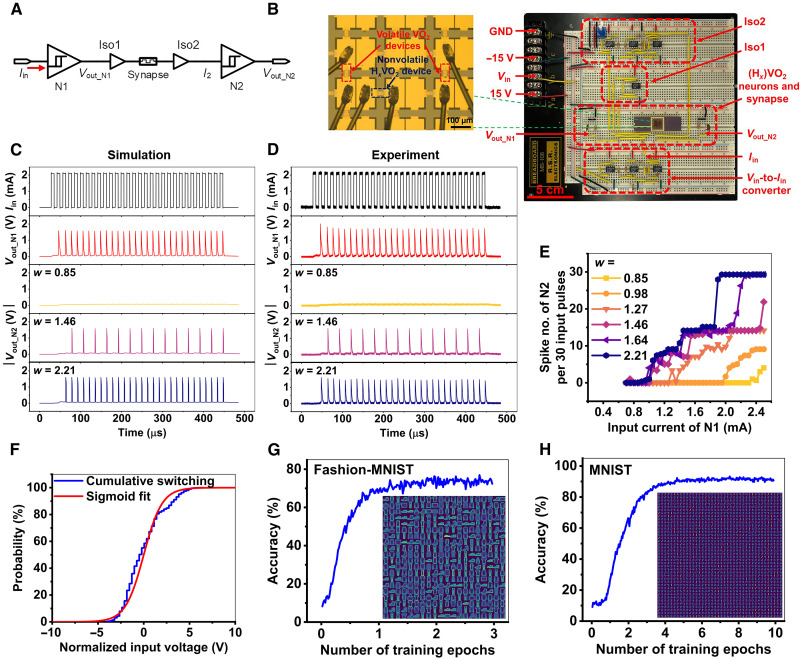
(H*_x_*)VO_2_ neural circuits and SNNs. (**A**) Schematic of a connected neural circuit for the feedforward excitation and inhibition motifs. A presynaptic neuron (N1), a synapse, and a postsynaptic neuron (N2) are connected in series with two isolators inserted between them. (**B**) Photograph of the neural circuit hardware. Inset: Neuromorphic chip with both volatile neuron-like and nonvolatile synapse-like (H*_x_*)VO_2_ devices. GND stands for ground. A voltage-to-current converter is used to convert voltage pulses (*V*_in_) from an arbitrary function generator into current pulses (*I*_in_) as the circuit input. (**C**) Simulated and (**D**) measured output voltage waveforms of N1 and N2 for different synaptic weights. The current pulse train injecting into N1 has an *A*_p_ of 2.1 mA, a *t*_p_ of 7 μs, and a *D*_p_ of 50%. (**E**) Measured input-current–dependent firing probability (defined by the output spike number per 30 input current pulses) of N2 under different synaptic weights. (**F**) Cumulative switching dynamics of the volatile VO_2_ neurons, which are fitted into sigmoidal stochastic switching neurons used in the network simulation. Training accuracy for (**G**) Fashion-MNIST and (**H**) MNIST over 3 and 10 training epochs, respectively. For Fashion-MNIST, we have a batch size of 16, while it is 32 for MNIST. For both datasets, each epoch consists of 60,000 training images. We obtained a final test accuracy of 73.22% on Fashion-MNIST and 92.1% on MNIST. Inset of (G) is the trained weight pattern for Fashion-MNIST. The network of 400 excitatory neurons was trained over the 60,000 training images for three epochs. Inset of (H) is the trained weight pattern for MNIST. The network of 1600 excitatory neurons was trained over the 60,000 training images for 10 epochs.

Through selective area doping, both volatile and nonvolatile (H*_x_*)VO_2_ devices can be integrated on a single chip (see Materials and Methods and fig. S29). Their interconnections allow the demonstration of feedforward excitation/inhibition neural circuits, which are fundamental micronetwork motifs. The circuit hardware primarily consists of a presynaptic neuron (N1), a postsynaptic neuron (N2), a synapse, and peripheral circuits (Fig. 4, A and B). The two neurons are connected in series through a synapse to control spiking signal transmission from N1 to N2 by adjusting the weight. To avoid detrimental voltage fluctuations due to signal interferences, operational amplifiers (op-amps) are introduced between neural components. Specifically, a voltage follower [i.e., isolator 1 (Iso1)] is placed between the N1 and the synapse (*38*), and an inverting amplifier plus a Howland current pump (*39*, *40*) for voltage-to-current conversion (i.e., Iso2) are installed between the synapse and the N2 (*41*). The synaptic weight (*w*) here is defined as the absolute value of the inverting amplifier’s gain (i.e., the ratio of the resistance across the op-amp over the effective synapse resistance) (fig. S30). By tuning the input current pulses, we can directly modulate the spikes emanating from N1. Because of the existence of Iso1, the downstream synapse has little impact on N1, showcasing similar spiking characteristics under different synaptic weights (fig. S31). However, before transferring into N2, the ejected spike train needs to be processed by the synapse, so the firing of N2 is closely related to the synaptic weight. As shown in Fig. 4 (D and E), with the decrease (increase) of synaptic weight, the firing probability of N2 decreases (increases), indicating that the N2 behaves like an inhibitory (excitatory) neuron. The feedforward excitation/inhibition neural circuits are further simulated using Simulation Program with Integrated Circuit Emphasis (SPICE). The neuronal behavior can be well reproduced using an equivalent circuit consisting of a threshold switch and a capacitor (fig. S32). The simulated spiking behaviors of N1 and N2 under different synaptic weights and input current pulses are in good agreement with the results measured in hardware (Fig. 4C and fig. S33). The consistency of simulation and measurement results augment the credibility of recognition tasks that are executed in the (H*_x_*)VO_2_ neural networks. In this work, Iso1 and Iso2 were inserted in the connection between one synapse and one neuron, but it is not necessary to reserve an exclusive isolator for each synapse-neuron pair when the connection scales up (i.e., between multiple synapses and one neuron). These isolators can be shared by serving as a part of neuron peripheral circuitry wherein multiplexers and demultiplexers are included between adjacent neural layers and multiple synapses are connected in parallel.

### (H*_x_*)VO_2_-based SNNs

To showcase the excitatory and inhibitory neural circuits using both volatile and nonvolatile (H*_x_*)VO_2_ devices in the context of network-level recognition tasks, an SNN was designed using the experimental data from the devices. SNNs use “spikes” and learning occurs by modulating the synaptic weights based on the relative timing of the spikes, known as spike timing–dependent plasticity (STDP) (*42*). The local, sparse, and unsupervised nature of STDP lends itself to low-power on-chip embedded intelligence applications. The network architecture for the SNN is shown in Fig. 1C. The network is made of volatile VO_2_ neurons connected by nonvolatile H*_x_*VO_2_ synapses. In hardware implementations, such a system can be mapped to a cross-array of synapse devices, terminating at neuronal devices. The stochastic switching of the neuron was sigmoidal in nature (Fig. 4F). The linear potentiation of the H*_x_*VO_2_ was used in simulating the synaptic conductance change. We trained the network on the MNIST (*26*) and Fashion-MNIST (*27*) datasets, each consisting of 10 classes of 28-by-28 pixel grayscale images of handwritten digits and fashion products, respectively. Both datasets used in the simulations were composed of 60,000 training samples and 10,000 test samples. The inputs to the network were Poisson spike trains, generated from the image pixel values. In addition, for the Fashion-MNIST, the dataset was filtered using the Sobel filter for edge detection before converting to spikes. The simulated networks consisted of an input layer of size 784 (equivalent to the dimensionality of the input data), an excitatory layer, and an inhibitory layer of 1600 each for MNIST and 400 each for Fashion-MNIST. All the neurons in the excitatory layer are connected to all the neurons in the input layer with excitatory connections. The neurons in the inhibitory layer are connected to one corresponding excitatory neuron with excitatory synapses and with inhibitory synapses to all the rest. The inhibitory connections achieve winner-take-all functionality in the SNN. The employment of the inhibitory connections greatly increases the generalization capability of the network, as shown in fig. S34. Note that only the excitatory connections from the input to the excitatory layer are tunable, while the other synaptic conductance remains static. Details of the network simulation can be found in Materials and Methods. After training, three independent test runs on the two datasets were performed. The simulated networks achieved an average test accuracy of 73.29% (SD: 0.09%) for Fashion-MNIST (Fig. 4G) and 92.26% (SD: 0.05%) for MNIST (Fig. 4H). These accuracies are in line with state-of-the-art unsupervised approaches for both datasets (*43*, *44*), demonstrating the efficacy of (H*_x_*)VO_2_ devices for scalable neuromorphic systems.

## DISCUSSION

Selective area electron doping is demonstrated for archetypal Mott oxide electronic devices using insulator-metal transitions. This process enables monolithic integration of homotypic volatile threshold firing neurons and nonvolatile synapses for memory, providing a platform to implement Mott neuromorphic hardware for brain-inspired computing electronics and neural systems emulation studies. In the long term, the approach presented here can be of use to various emerging semiconductors that require heavy concentrations of dopants in device technologies.

## MATERIALS AND METHODS

### VO_2_ thin-film deposition

Before deposition, 10-by-10 mm^2^ c-plane sapphire substrates were cleaned through an ultrasonic bath of toluene, acetone, and isopropyl alcohol in sequence. The 50-nm-thick VO_2_ thin films were deposited using an ultrahigh vacuum magnetron sputtering system from AJA International Inc. During deposition, a V_2_O_5_ ceramic target was used in the deposition powered with a radio frequency source at 100 W. The substrate temperature was 650°C. The chamber pressure was set at 5 mTorr of the Ar/O_2_ mixture with a ratio of 99.9/0.1. The deposition rate was 0.6 nm/min. Before material characterizations, the H*_x_*VO_2_ samples were annealed at 200°C in a homebuilt furnace filled with a forming gas (5% H_2_ balanced by 95% Ar) for 30 min.

### (H*_x_*)VO_2_ device fabrication

Both volatile (TiAu + TiAu) and nonvolatile (TiAu + Pd) VO_2_ devices were fabricated on a single sapphire substrate. After substrate cleaning and 50-nm-thick VO_2_ thin-film deposition, the thin film was patterned using argon ion milling, during which SPR-220-3.5 photoresist served as a mask layer. Next, Ti (10 nm)/Au (100 nm) contact pads were formed through photolithography with LOR-3A and S1813 photoresists, electron beam evaporation (Ti deposition came first), and liftoff in Remover PG (Kayaku Advanced Materials,.Inc) at 80°C. To define the submicrometer channels, electron beam lithography was used in the next steps, where 495 poly(methyl methacrylate) (PMMA) A4 and 950 PMMA A4 were used as photoresists. Ti (10 nm)/Au (50 nm) stripes in the TiAu + TiAu and the TiAu + Pd devices were patterned by electron beam evaporation and liftoff in acetone at room temperature. The 50-nm-thick Pd electrode strips in the TiAu + Pd devices were patterned by repeating the processes for the TiAu electrode strips. The fabricated devices were annealed at 100°C in the forming gas with a flow rate of 50 standard cubic centimeter per minute to selectively hydrogenate the TiAu + Pd VO_2_ device. For the feedforward excitation/inhibition neural circuits, the volatile and nonvolatile devices on the same chip were interconnected by further wire bonding to a chip carrier.

### Electrical measurements

The electrical performance of (H*_x_*)VO_2_ device was characterized at room temperature by a Keithley 4200A-SCS parameter analyzer with ultrafast pulse measure units (4225-PMU) and remote preamplifier/switch modules (4225-RPM), which can apply submicrosecond electric pulses. For all electrical measurements, the TiAu electrode was connected to the ground. For (H*_x_*)VO_2_ neural circuits, in situ *I*-*V* measurement setups were designed and customized. The pulse trains applied across the circuits were generated by an arbitrary function generator (Tektronix AFG31000 Series), while the voltage signals were monitored using an oscilloscope (Tektronix DSOX4154A). A high-speed voltage-to-current converter was connected to the arbitrary function generator for applying microsecond current pulses to the (H*_x_*)VO_2_ neural circuits. For the neuronal behavior, the TiAu + TiAu VO_2_ device was connected in series with a 200-ohm load resistor (*R*_L_) and a 4.4-nF capacitor (*C*_M_) in parallel. The voltage drops across the VO_2_ device (*V*_VO2_), and the *R*_L_ (*V*_out_) were acquired from the oscilloscope. The current passing through the TiAu + TiAu VO_2_ device was calculated with the equation *I* = *V*_out_/*R*_L_. For testing the individual neuron behavior in connected neuron-synapse-neuron circuits, customized isolators were used between the three components to avoid signal interferences among these devices, which might adversely affect signal propagation throughout the circuit.

### Simulation methodology for neural circuits

Both single neuronal component and neural circuit behaviors were simulated with LTspice. The TiAu + TiAu VO_2_ device was modeled as a threshold switch highlighted by a red dash box, as shown in fig. S32A. The off-state resistance (*R*_H_) of the threshold switch was constant, while the on-state resistance (*R*_L_) was controlled by the voltage across the threshold switch (*V*_in_ − *V*_out_). Meanwhile, a minimum *R*_L_ (i.e., *R*_min_) was imposed through the transition voltage (*V*_sim_). If *V*_in_ − *V*_out_ > *V*_sim_, then the on-state resistance would be directly assigned with *R*_min_. If *V*_in_ − *V*_out_ < *V*_sim_, then the relationship between *R*_L_ and *V*_in_ − *V*_out_ follows an empirical formula acquired through fitting the *I*-*V* characteristic in fig. S32B; i.e., *R*_L_ = 1000/[α + β × (*V*_in_ − *V*_out_) + γ × (*V*_in_ − *V*_out_)^2^]. The parameters for single neuronal component simulations are listed in table S1 and fig. S32D. In the neural circuit simulation, a programmable resistor serving as the synapse was connected between Iso1 and Iso2. Because pre- and postsynaptic neurons showed stochasticity, their parameters used in the simulation (table S2) were individually adjusted to fit their own behaviors better.

### Simulation methodology for network-level recognition tasks

SNNs use bioplausible local learning mechanisms motivated by the propagation of electric impulses in the biological brain, often referred to as STDP (*45*, *46*). In STDP, the synaptic weights are modified in an exponential manner based on the relative timing difference between pre- and postsynaptic neuronal activities. The SNN implementation was done using a modified version of BindsNET (*47*), an open-source PyTorch-based framework. The network architecture was adopted from (*43*) for both the MNIST and Fashion-MNIST datasets. The stochastic neuron model with the VO_2_ neuron dynamics was used. The linear potentiation of the H*_x_*VO_2_ synapse with four-bit discretization was taken. The weight dynamics is computed using synaptic traces (*48*), and the weight update rule is characterized by

$$\text{Δ}w=\left\{ \begin{array}{c} \eta_{\mathrm{post}}\times \chi_{\mathrm{pre}}\;\text{on postsynaptic spike}\\ -\eta_{\mathrm{pre}}\times \chi_{\mathrm{post}}\;\text{on presynaptic spike} \end{array}\right.$$ (1)

Here, χ_pre_ and χ_post_ are the pre- and postsynaptic traces, respectively, and are set to 1 whenever the pre- or postsynaptic neurons fire and decay exponentially to 0 with decay time constant τ_trace_. η_post_/η_pre_ represent the pre- and postsynaptic learning rates. To mimic quantized weight updates typically observed in such device technologies (*49*), the weights in the network-level simulations were quantized using

$$w_{q}=\frac{\mathrm{round}[(2^{N}-1)\times w]}{2^{N}-1}$$ (2)

Here, *w* and *w*_q_ are the nonquantized and quantized weights, respectively, and *N* is the quantization precision. Lateral inhibition is implemented using the inhibition layer and its inhibitory connections. To stop a single neuron from dominating the learning process, homeostasis was implemented by adaptively scaling the input to the stochastic neuron. The network parameters are given in table S3.

## Supplementary Materials

### This PDF file includes:

Supplementary Text Figs. S1 to S34 Tables S1 to S3 References
