# Supplementary material for: Selective area doping for Mott neuromorphic electronics
Source: Sci Adv. 2023 Mar 17;9(11):eade4838. doi: 10.1126/sciadv.ade4838 (PMC10022892; doi:10.1126/sciadv.ade4838)
Supplement: Supplementary file 1 — Supplementary Text Figs. S1 to S34 Tables S1 to S3 References [file sciadv.ade4838_sm.pdf]

Science Advances
AAAS

Supplementary Materials for

Selective area doping for Mott neuromorphic electronics

Sunbin Deng *et al.*

Corresponding author: Sunbin Deng, deng256@purdue.edu; Tae Joon Park, park1080@purdue.edu; Shriram Ramanathan, shriram@purdue.edu

Sci. Adv. **9**, eade4838 (2023)
DOI: 10.1126/sciadv.ade4838

This PDF file includes:

Supplementary Text
Figs. S1 to S34
Tables S1 to S3
References

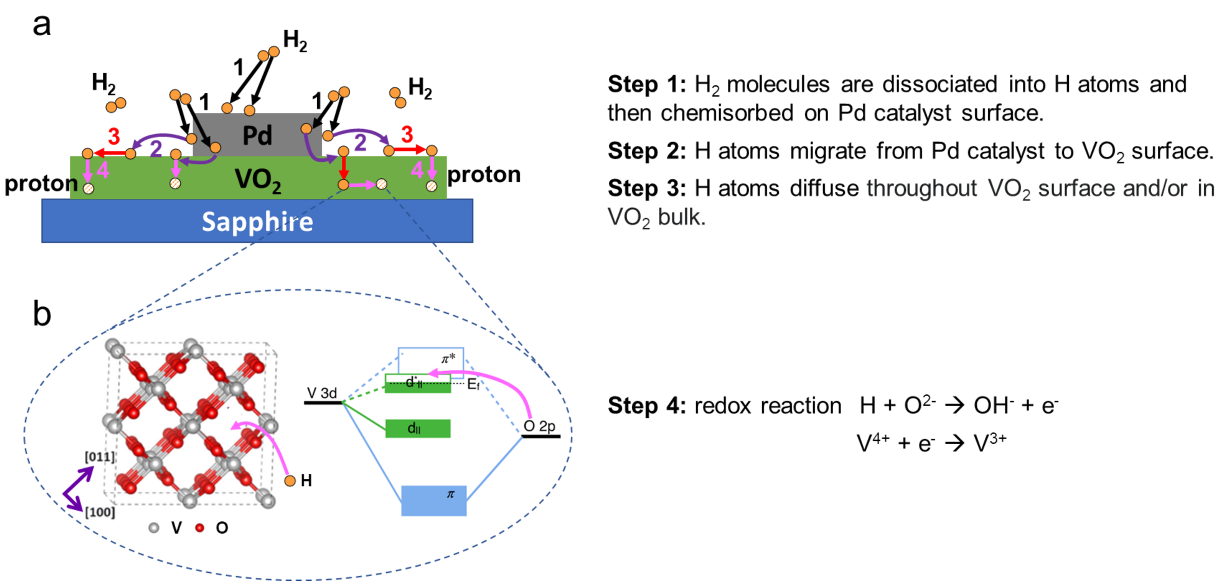

Fig. S1. Schematic of catalytic hydrogen doping of VO_2. (a) Pd-catalyst-assisted hydrogen spillover process. (b) Redox reactions through the hydrogenation modulate the electronic band structure of VO_2 (*33*) and create a metallic state.

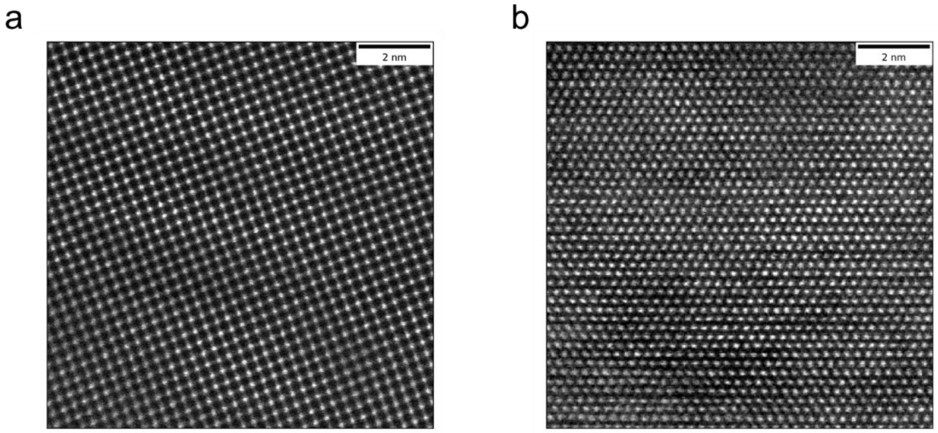

Fig. S2. High-resolution STEM electron micrographs. The local lattice of (a) VO_2 and (b) H_xVO_2 crystalline grains are shown.

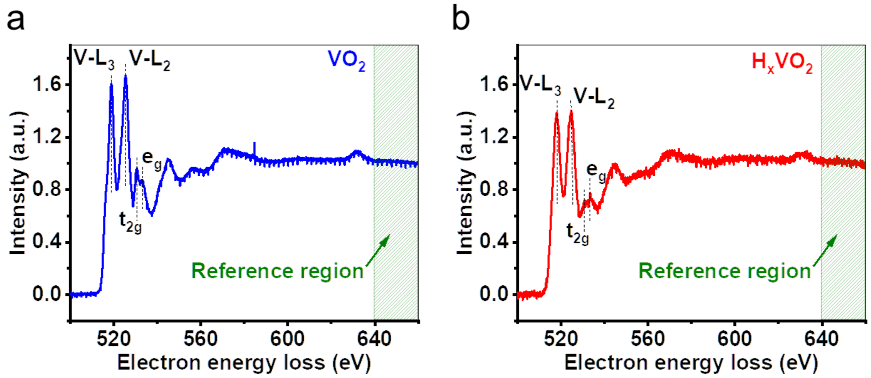

Fig. S3. EELS spectra from (a) VO_2 and (b) H_xVO_2 region. The averaged intensity level between 640 eV and 660 eV was used as a reference to normalize the spectra.

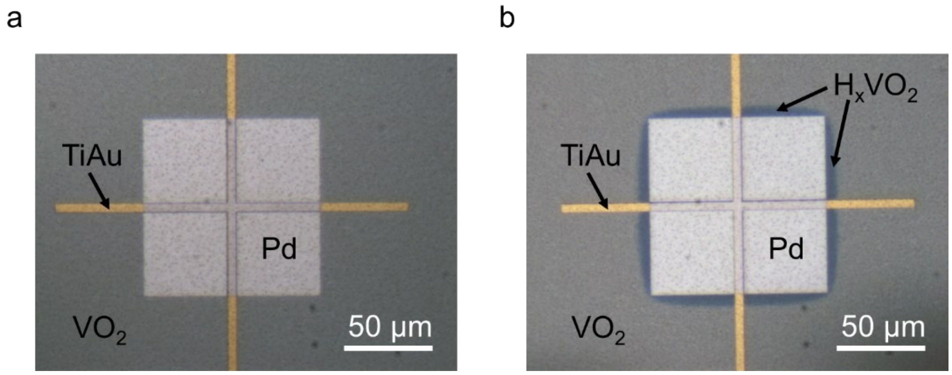

Fig. S4. Optical microscope images of (H_x)VO_2 with Pd and TiAu electrodes. The image (a) before and (b) after the hydrogenation (i.e., annealing at 200°C in the forming gas for 30 mins). The thickness of the Pd, Ti, Au, and VO_2 are 50 nm, 10 nm, 50 nm, and 50 nm, respectively. The region around the Pd square becomes bluish due to the formation of H_xVO_2 after the hydrogenation. In contrast, the region near the crossed TiAu electrodes remains the same (grey) (*50*), indicating that TiAu is inert during the catalytic hydrogenation of VO_2.

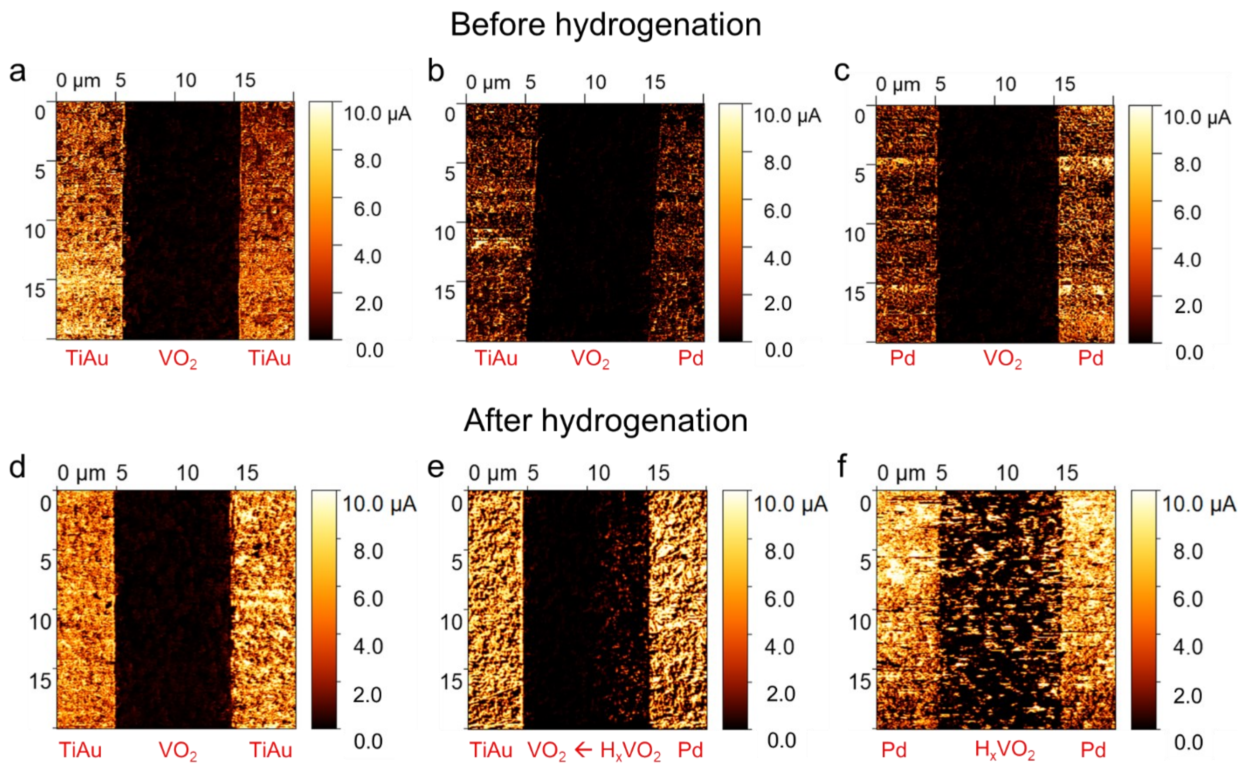

Fig. S5. C-AFM images of two-terminal (H_x)VO_2 devices with different electrode combinations. (a) TiAu+TiAu device, (b) TiAu+Pd device, and (c) Pd+Pd device were probed before the hydrogenation. (d) TiAu+TiAu device, (e) TiAu+Pd device, and (f) Pd+Pd device were probed after the hydrogenation.

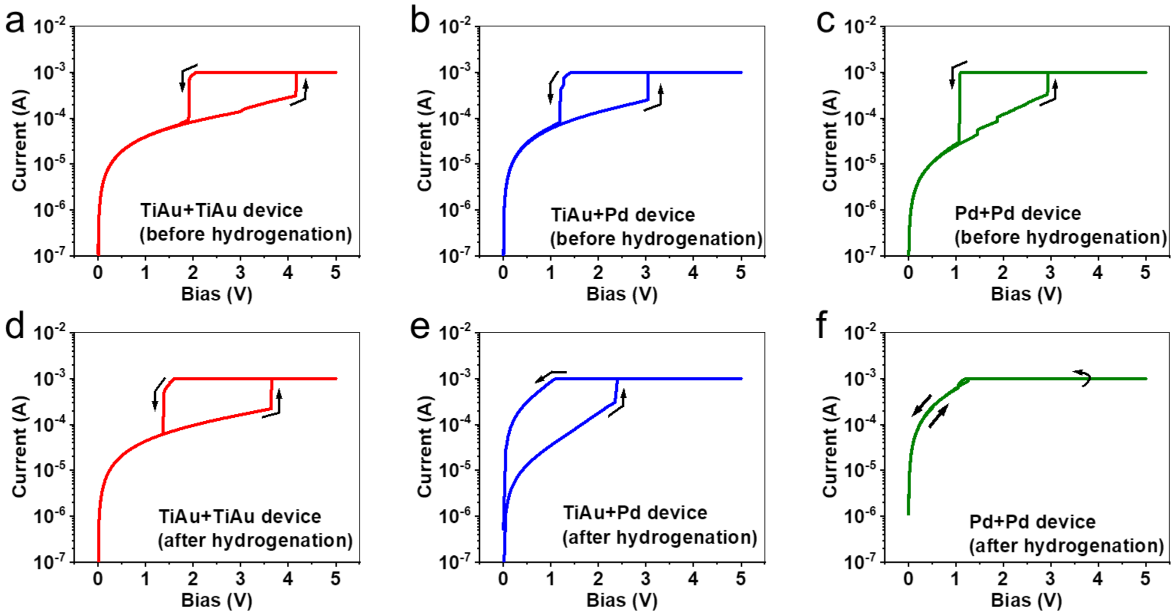

Fig. S6. I-V curves of two-terminal (H_x)VO_2 devices with different electrode combinations. (a) TiAu+TiAu device, (b) TiAu+Pd device, and (c) Pd+Pd device were swept before the hydrogenation. All the devices show threshold switching. (d) The TiAu+TiAu device, (e) the TiAu+Pd device, and (f) the Pd+Pd device were measured after the hydrogenation. The TiAu+TiAu device retains the pristine VO_2 channel and still shows the volatile threshold switching, but the Pd+Pd device with the H_xVO_2 channel behaves like a resistor. The TiAu+Pd device exhibits a non-volatile switching due to proton migration and redistribution across the channel.

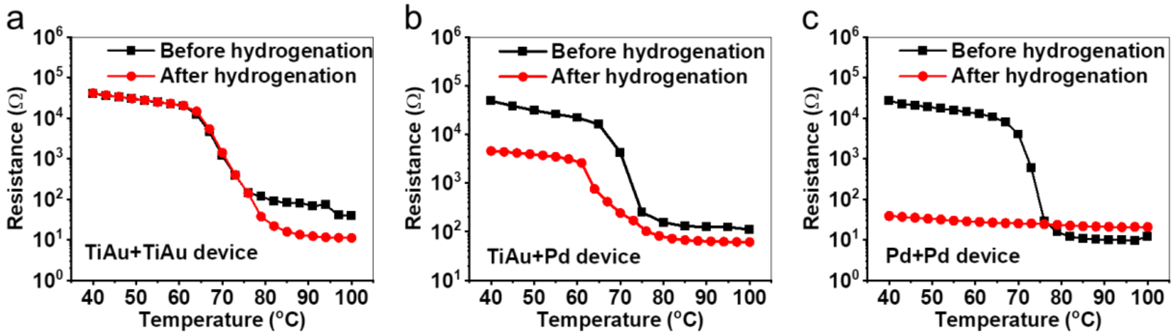

Fig. S7. Temperature-dependent resistance of $(H_x)VO_2$ devices with different electrode combinations. (a) The TiAu+TiAu device, (b) the TiAu+Pd device, and (c) the Pd+Pd device. Before the hydrogenation, all the devices show the thermally driven insulator-metal transition (T-IMT) phenomenon at a temperature ($T_{critial}$) of ~67°C, and the resistance decreases by nearly three orders of magnitude. After the hydrogenation, the TiAu+TiAu device exhibits a similar R-T curve, while the T-IMT of VO_2 in the Pd+Pd device is greatly suppressed. The TiAu+Pd device shows a lower ground-state resistance because its channel is partially doped with hydrogen ions.

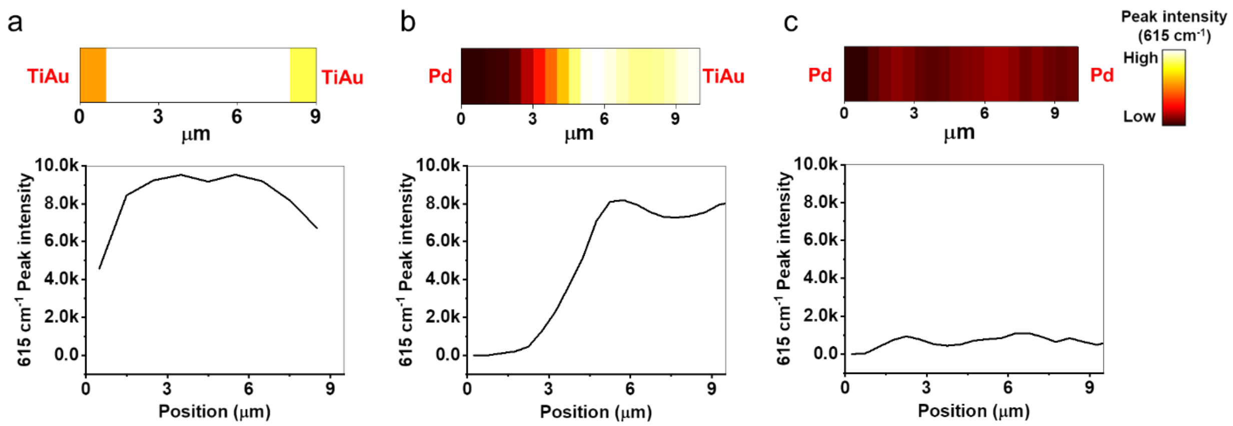

Fig. S8. Line-scan of Raman spectra across the channels of (H_x)VO_2 devices with different electrode combinations. (a) The TiAu+TiAu device, (b) the TiAu+Pd device, and (c) the Pd+Pd device. Raman peak of VO_2 is fixed at 615 cm^{-1}. All devices were treated under the same hydrogenation condition. High Raman peak intensity at 615 cm^{-1} for the TiAu+TiAu device was observed, while the peak intensity for the Pd+Pd device was almost suppressed. Line-scan of Raman spectra for the TiAu+Pd device shows gradients across the channel, indicating selective hydrogenation of VO_2 near the Pd electrode (*36*).

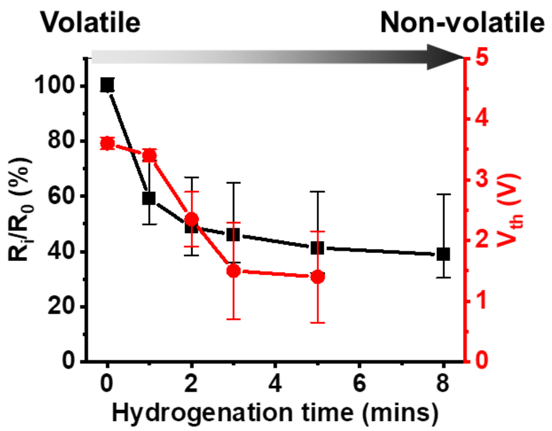

Fig. S9. Hydrogenation-time-dependent resistance changes and threshold voltage of the TiAu+Pd device. R_0 and R_i denote the initial resistance and the resistance after the hydrogenation, respectively. Hydrogenation was performed in forming gas at 100°C. After the hydrogenation for over 5 mins, the device shows no threshold switching, and V_{th} is not observed.

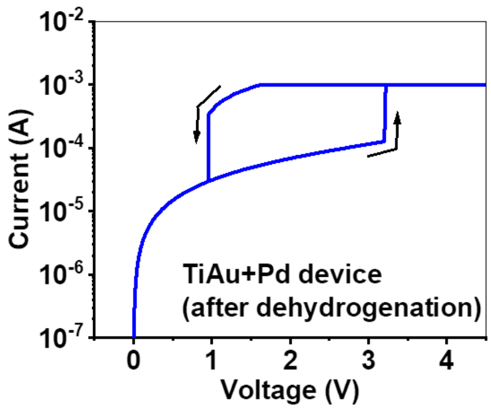

Fig. S10. I-V curve of the TiAu+Pd device after dehydrogenation. The dehydrogenation process was performed at 200°C in argon gas for 1 hour. The channel returned to the pristine monoclinic VO_2 showcasing the E-IMT feature (*23*).

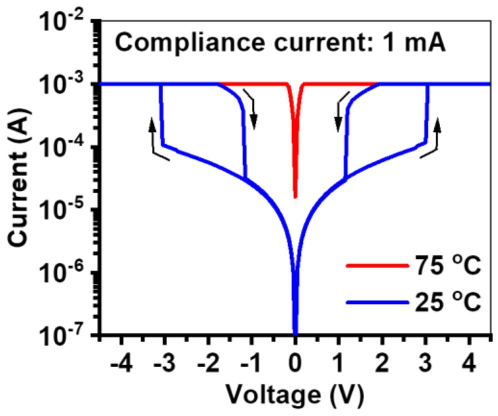

Fig. S11. I-V curves from the TiAu+TiAu devices were measured at 25°C and 75°C. The device stays in the metallic state and does not show volatile switching at 75°C. It indicates the volatile switching feature is indeed due to the metal-insulator transition of VO_2. To protect the device, the compliance current was set to 1 mA in the measurement.

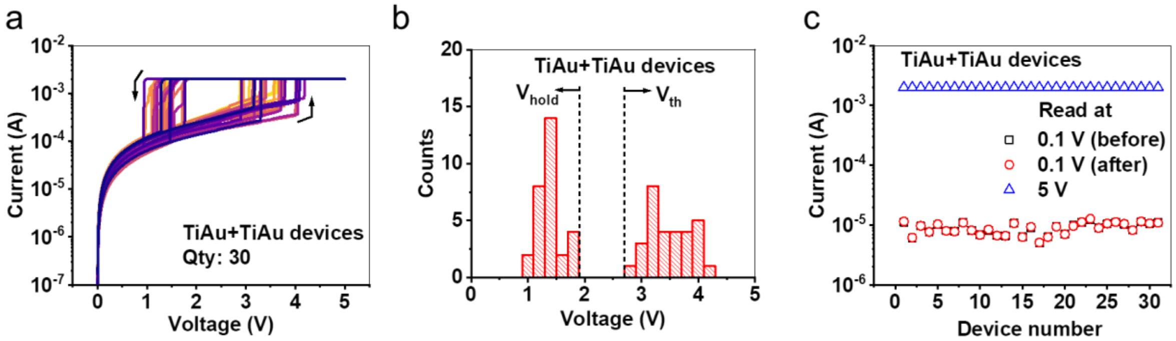

Fig. S12. Device-to-device variation of the TiAu+TiAu devices after the selective hydrogenation. (a) I-V curves of 30 devices fabricated on a single sapphire substrate (size: 10 × 10 mm^2). The DC sweeps are applied (0 V → 5 V → 0 V) with a compliance current of 2 mA. (b) Device-to-device distributions of threshold voltage (V_{th}) and holding voltage (V_{hold}). The average V_{th} and V_{hold} are 3.50 V and 1.38 V, respectively. The standard deviations of V_{th} and V_{hold} are 0.39 V and 0.22 V, respectively. (c) Device-to-device distributions of on- and off-state current read at 5 V and 0.1 V. Before and after the hydrogenation, off-states read at 0.1V remain the same. Current compliance of 2 mA was applied for device protection in the measurement. A clear separation between the high- and the low-resistance state is seen.

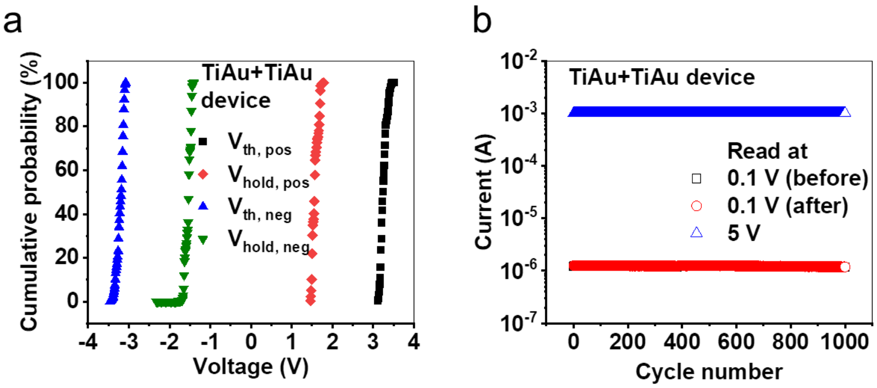

Fig. S13. Cycle-to-cycle variation of the TiAu+TiAu devices after the selective hydrogenation. (a) Cumulative probability of threshold voltage and holding voltage in both positive and negative bias polarities. 1000 consecutive DC sweeps are applied (0 V → 4 V → 0 V → -4 V → 0 V) with a compliance current of 1 mA. The average V_{th} and V_{hold} in positive and negative polarities are 3.27 V, 1.58 V, -3.21 V, and -1.54 V, respectively. The standard deviations of V_{th} and V_{hold} in positive and negative polarities are 0.077 V, 0.079 V, 0.081 V, and 0.073 V, respectively. (b) Cycle-to-cycle distributions of on- and off-state current read at 5 V and 0.1 V. Current compliance of 1 mA was applied for device protection in the measurement.

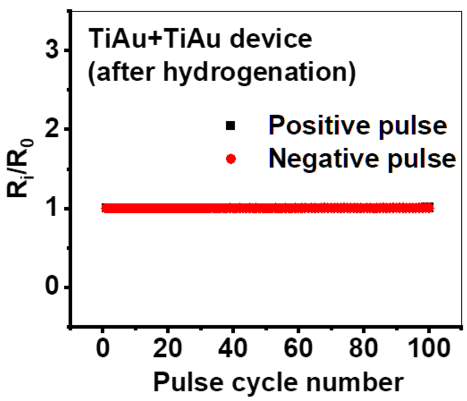

Fig. S14. Relative resistance changes upon multi-cycle electric pulse stimuli applied to the TiAu+TiAu device after the selective hydrogenation. R_0 and R_i denote initial device resistance and measured device resistance after every pulse stimulation. Periodic positive pulse (6 V/µm, 3 µs) and the negative pulse (-6 V/µm, 260 ns) are applied to the device. The TiAu+TiAu device shows no resistance change, indicating reproducible threshold switching.

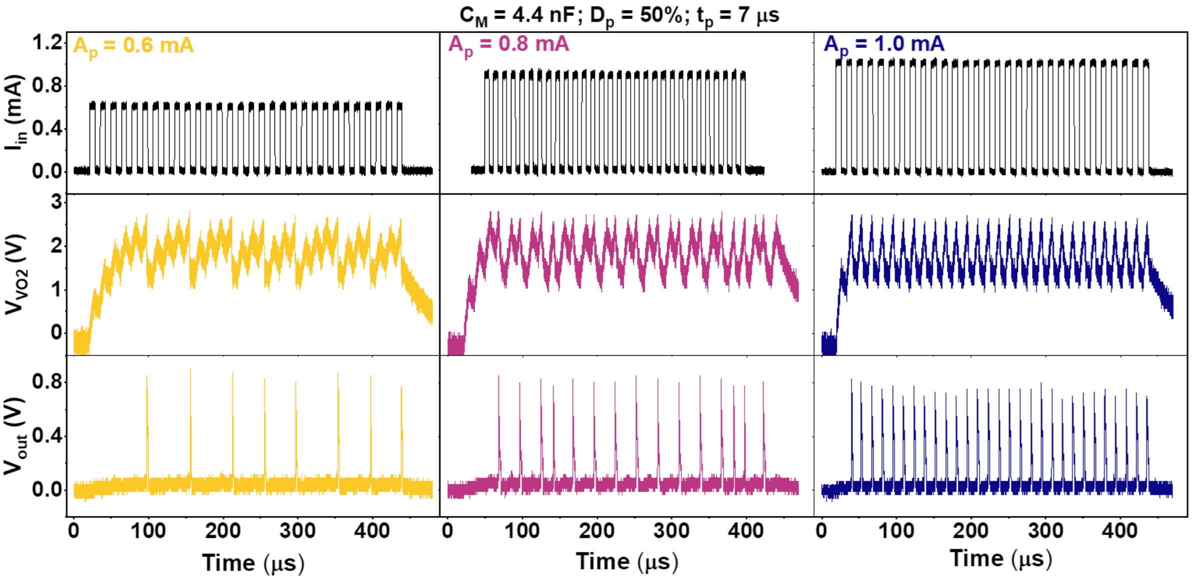

Fig. S15. Waveforms of the LIF neurons from the TiAu+TiAu devices after the selective hydrogenation. The LIF neurons are triggered by current pulse trains of different current amplitudes. The firing rate of LIF neurons increases with current amplitude. V_{out} is the membrane potential. At the reset stage of the LIF neurons, V_{out} drops to near zero after each firing event, whereas V_{VO2} does not necessarily return zero and only drops to the V_{hold} level (here it is ~1.4 V) for the TiAu+TiAu devices.

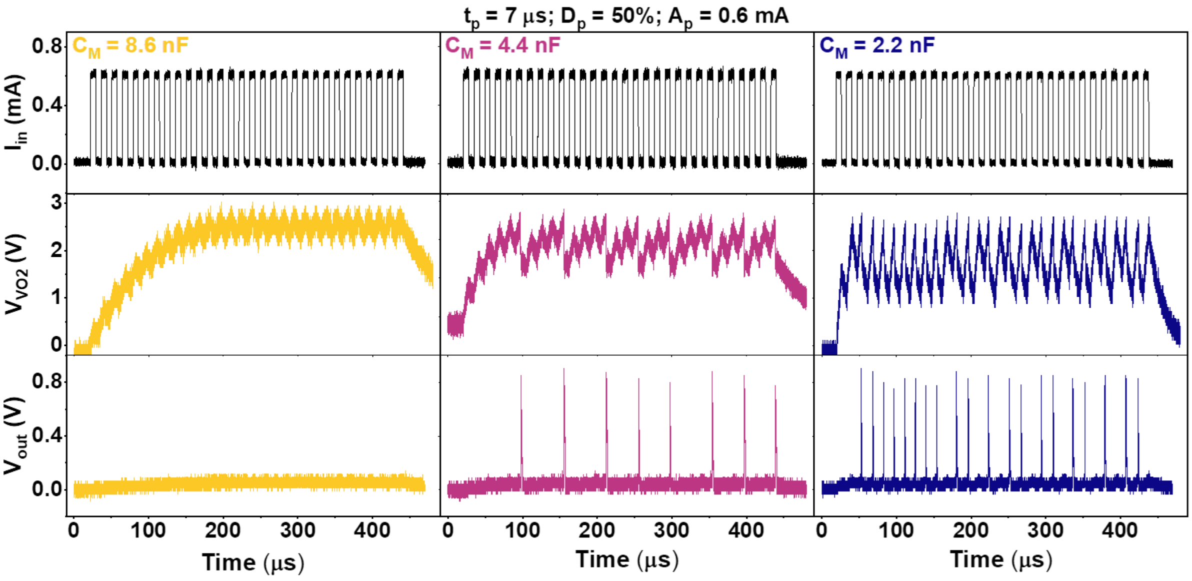

Fig. S16. Waveforms of the LIF neurons from the TiAu+TiAu devices after the selective hydrogenation. The LIF neurons are connected in parallel with different capacitors. As the capacitance value decreases, the spiking rate of LIF neurons increases.

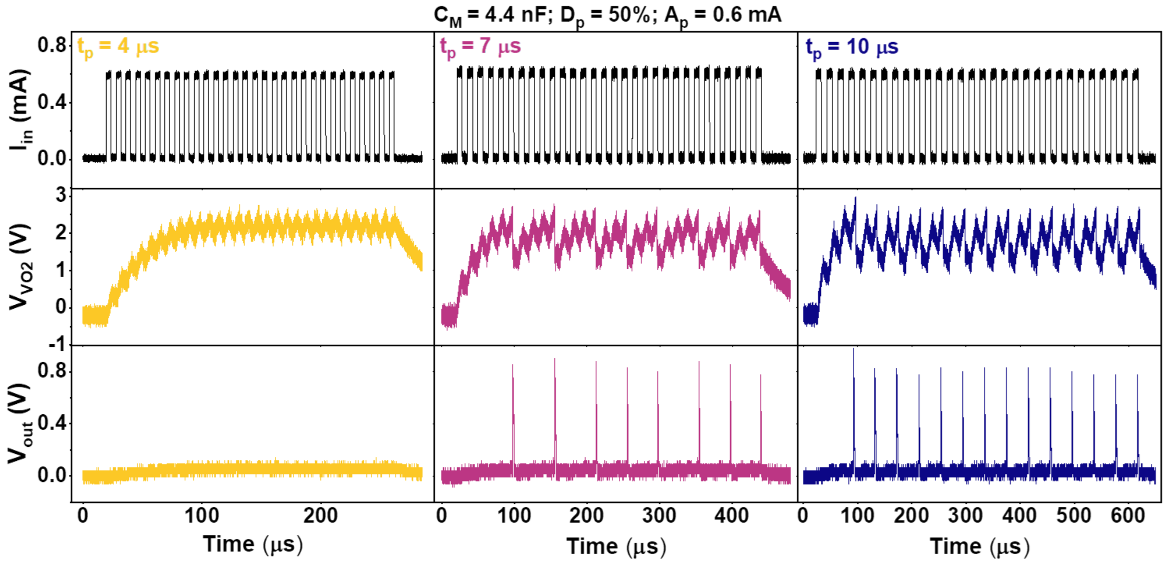

Fig. S17. Waveforms of the LIF neurons using the TiAu+TiAu devices after the selective hydrogenation. The LIF neurons are triggered by current pulse trains with different pulse widths. As the applied pulse width increases, the firing rate of LIF neurons increases.

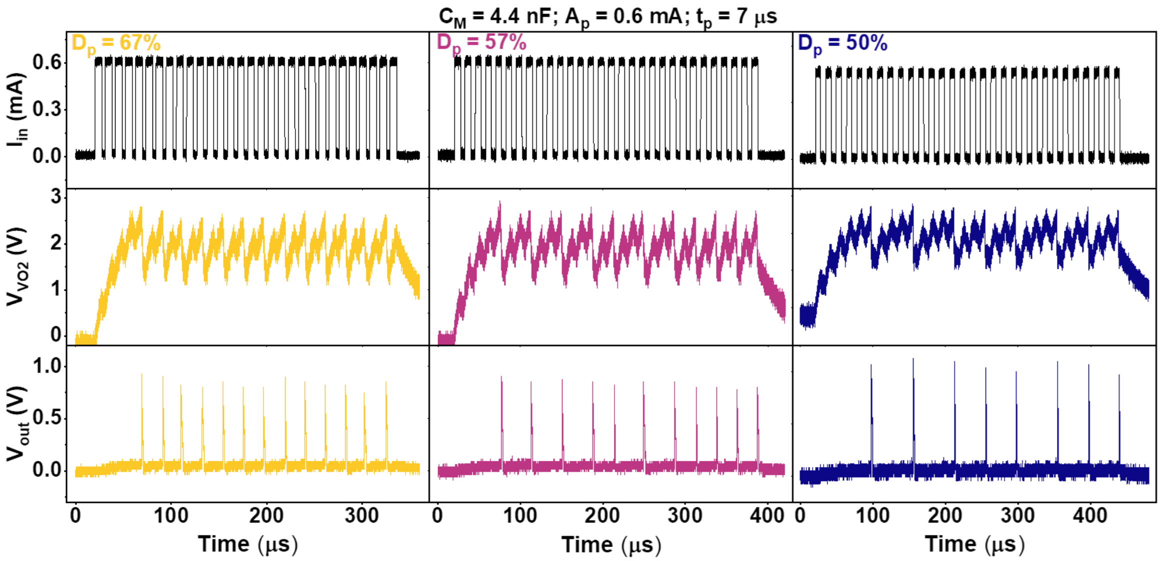

Fig. S18. Waveforms of the LIF neurons using the TiAu+TiAu devices after the selective hydrogenation. The LIF neurons are triggered by current pulse trains with different duty cycles. As the duty cycle decreases, the firing rate of LIF neurons also decreases.

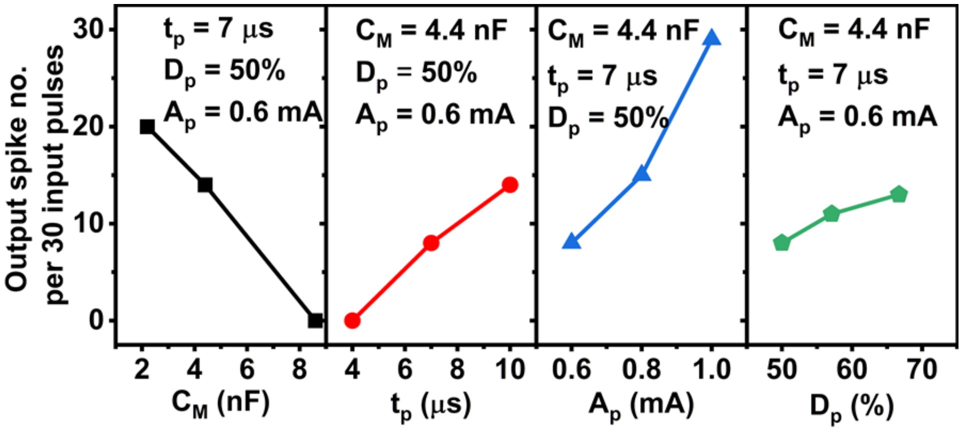

Fig. S19. Controlled output firing response of the neuronal component depending on C_M, t_p, A_p, and D_p. A higher C_M delays neuronal firing. In contrast, stronger pulse inputs with a larger t_p, A_p, or D_p increase the firing.

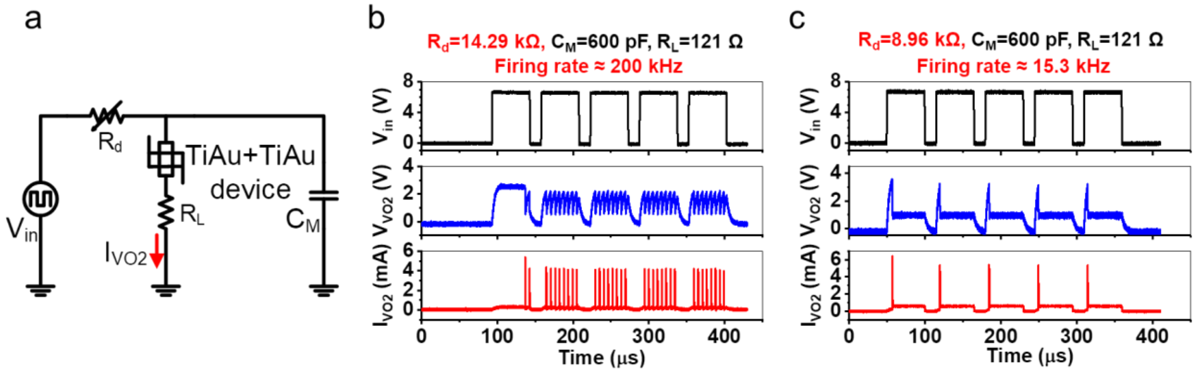

Fig. S20. LIF neuron triggered by voltage pulses. (a) Circuit diagram of the LIF neuron using the TiAu+TiAu device and voltage pulse inputs. (b) Waveforms of the LIF neuron with a voltage-divider resistor (R_d) of (b) 14.29 kΩ and (c) 8.96 kΩ. The neuron connected with R_d of 14.29 kΩ has a firing rate of ~200 kHz, while the one connected with R_d of 8.96 kΩ has to fire at a lower frequency of ~15.3 kHz because the TiAu+TiAu device does not have the load line well intersecting with its I-V curve in the negative differential resistance (NDR) region (*51*). The plateaus of V_{VO2} and I_{VO2} in (c) indicate that the TiAu-TiAu device remains in the conducting state after a firing event.

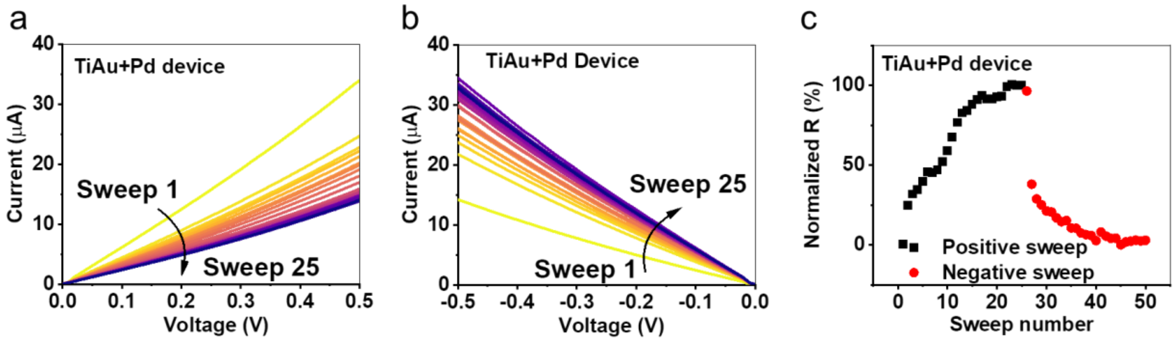

Fig. S21. Consecutive I-V sweeps of the hydrogenated TiAu+Pd devices. Consecutive I-V sweeps with (a) positive and (b) negative voltages applied to the devices, showing gradual changes in the measured currents. (c) Normalized device resistance after every sweep number. The device resistance increases (decreases) continuously after positive (negative) sweeps, showing analog synaptic weight updates.

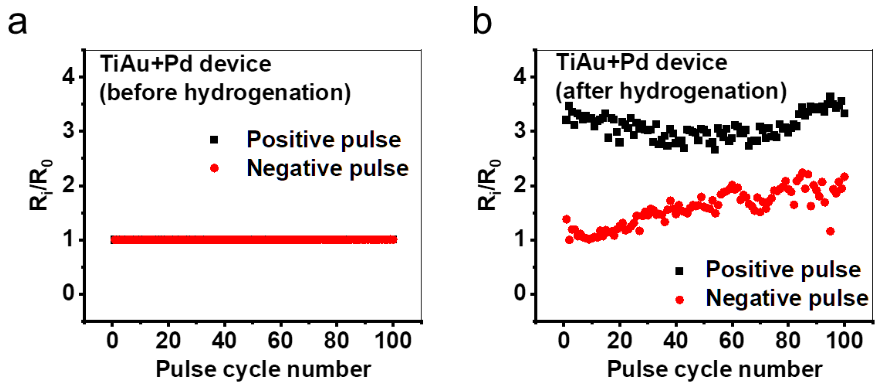

Fig. S22. Resistance response upon electric pulse stimuli applied to the TiAu+Pd devices. The resistance response of the devices (a) before and (b) after the hydrogenation. The positive pulse (6 V/μm, 3 μs) and the negative pulse (-6 V/μm, 260 ns) are applied alternately. After each pulse was applied, the resistance of the device was measured. Before the hydrogenation, the device does not show resistive switching. After the hydrogenation, the device shows non-volatile resistance changes.

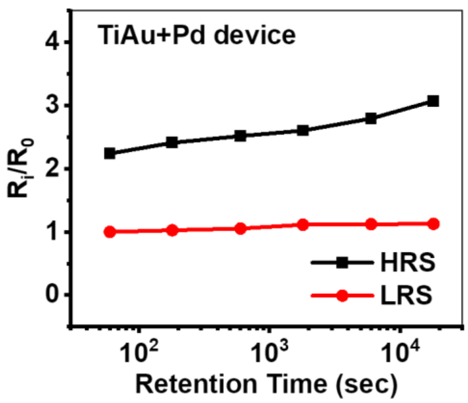

Fig. S23. Retention test of the hydrogenated TiAu+Pd device. The high-resistance state (HRS) and the low-resistance state (LRS) are programmed after the positive pulse (10 V/µm, 1 µs) and the negative pulse (-10 V/µm, 1 µs) are applied, respectively. Programmed states are stable up to 10^4 s.

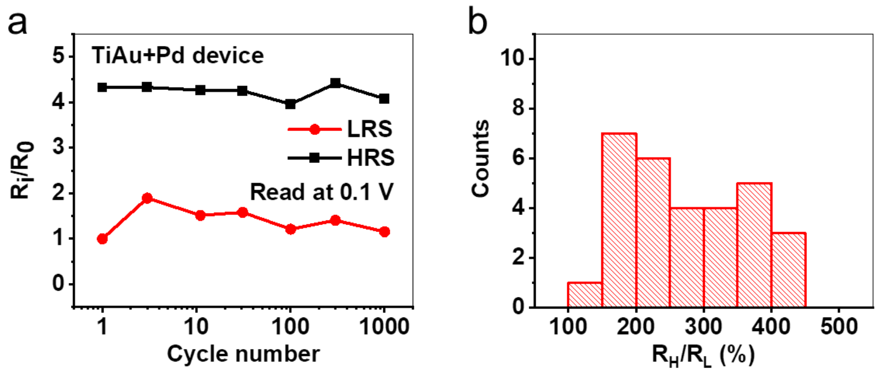

Fig. S24. Variation in the hydrogenated TiAu+Pd devices. (a) Cycle-to-cycle variation. The device can be programmed to the HRS and the LRS after 1000 consecutive I-V sweeps. Both the HRS and the LRS read at 0.1 V. (b) Device-to-device variation. The on-off ratio (R_H/R_L) among 30 devices fabricated on a single 10 × 10 mm^2 sapphire substrate ranges from 142% to 426% with an average value of 276% and a standard deviation of 0.86.

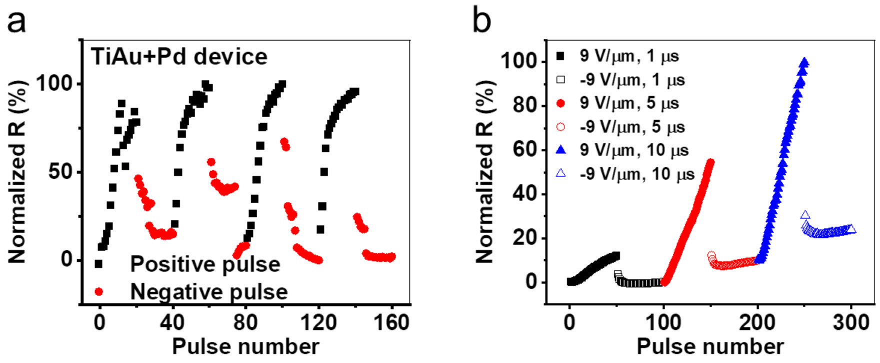

Fig. S25. Resistance potentiation and depression of the hydrogenated TiAu+Pd device. (a) The normalized resistance changes of the device upon 20 consecutive positive pulses (6 V/μm, 1 μs) followed by 20 consecutive negative pulses (-6 V/μm, 1 μs). Consecutive positive and negative pulses were repeated 4 times. The potentiation and depression processes are reproducible. (b) The normalized resistance changes of the device upon 50 consecutive positive pulses (9 V/μm) followed by 50 consecutive negative pulses (-9 V/μm). The pulse width varies from 1 μs to 10 μs. The device resistance changes depending on the applied pulse width. The discontinuity between the last potentiation state and the first depression state could be related to the asymmetry of proton concentration in the channels. Due to Pd-catalyst-assisted hydrogen spillover, there are a larger number of protons in the region near Pd. With the gradual reduction of protons at the Pd/VO_2 interface, the channel resistance continuously increases. When the external electric field switches to opposite polarity during the depression, the protons start to migrate back toward Pd. Because of the differences in the local concentration of protons near the Pd electrode, there could be a slight asymmetry in the drift characteristics for the two voltage polarities.

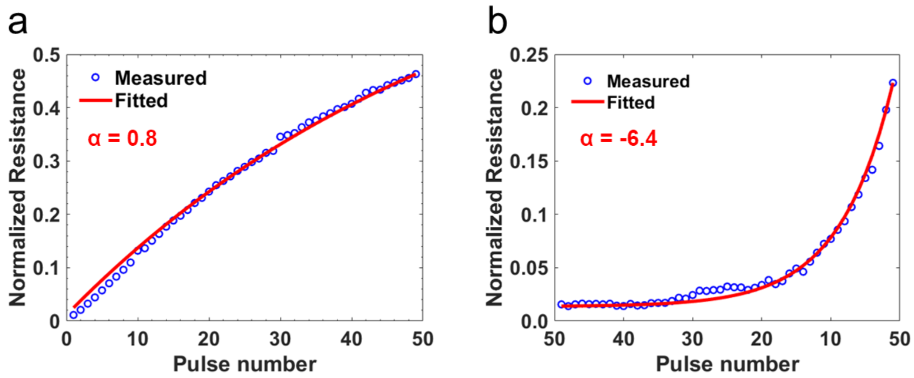

Fig. S26. Pulse number dependence of normalized resistance in the hydrogenated TiAu+Pd device. Extraction of nonlinear factors (*37*) in the resistance (a) potentiation and (b) depression. The positive and negative pulse conditions are (8 V/μm, 1 μs) and (-8 V/μm, 1 μs), respectively.

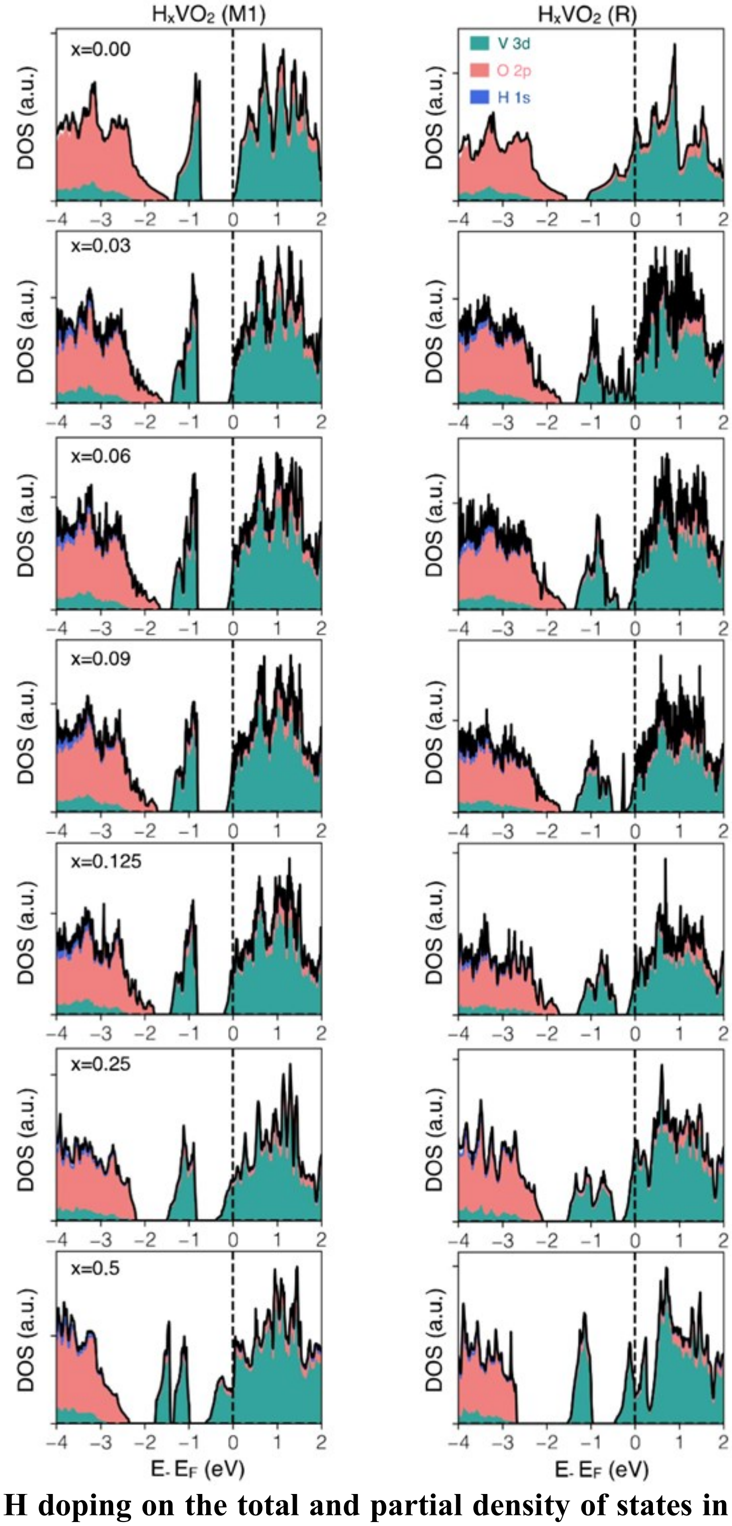

Fig. S27. Effect of H doping on the total and partial density of states in pseudo-monoclinic H_xVO_2 (M1) and pseudo-rutile H_xVO_2 (R) phases.

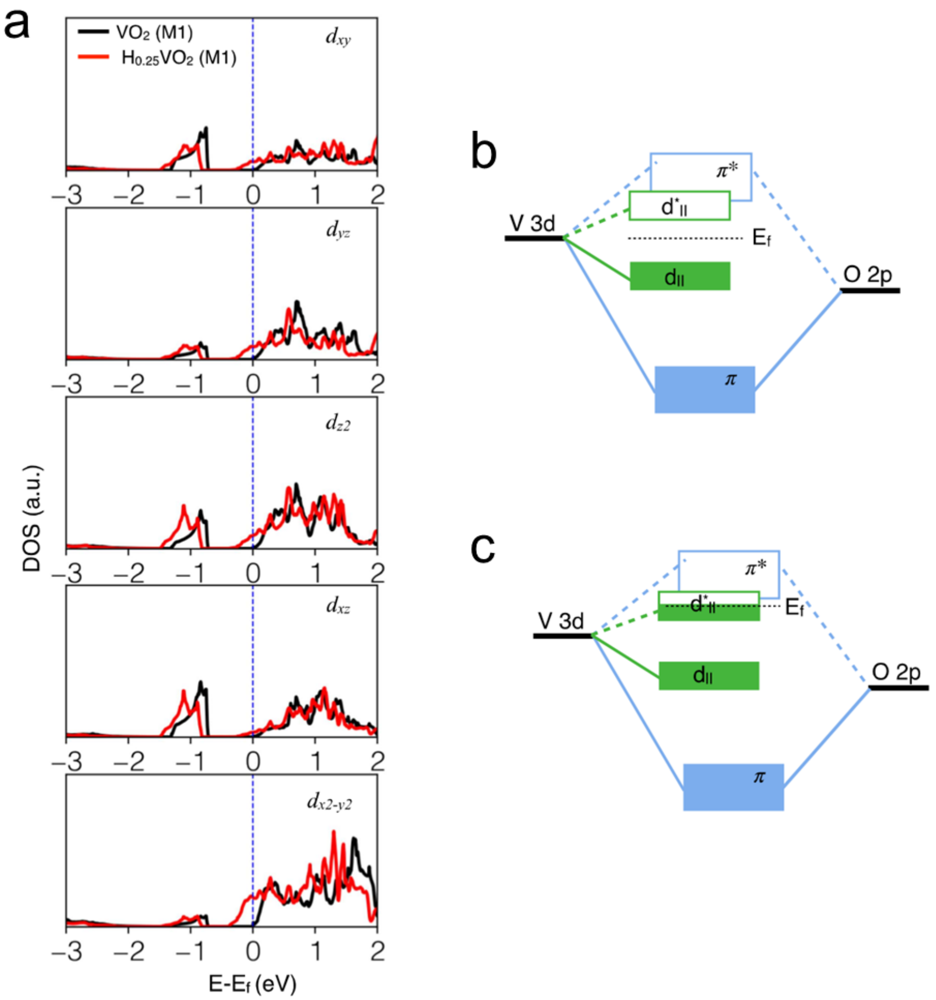

Fig. S28. Effect of H dopant on the resistance of the M1 phase. (a) Comparison of the PDOS of V 3d orbital between VO_2 (M1) and H_xVO_2 (M1). Schematics of molecular orbitals for (b) VO_2 (M1) and (c) H_xVO_2 (M1). The V atoms in VO_2 have an octahedral coordination with O atoms. According to crystal field splitting, the atomic d orbitals in an octahedral coordination geometry are split into two degenerate orbitals, i.e., low-energy t_{2g} states and high-energy e_g states. The components of doubly generated e_g are d_{z2} and d_{x2-y2} ($d_{||}$) whereas the components of triply generated t_{2g} states are d_{xy}, d_{yz}, and d_{xz}. In the case of VO_2 (M1) phase, the Fermi level located between the edge states of $d_{||}$ and $d_{||}$*/π* orbitals results in a gap of ~0.6 eV. Upon the addition of H, the $d_{||}$*/π* orbitals are partially filled, which results in metallic states. The same is also reflected in the shifting down of the occupancy of d_{x2-y2} ($d_{||}$) orbital upon H addition.

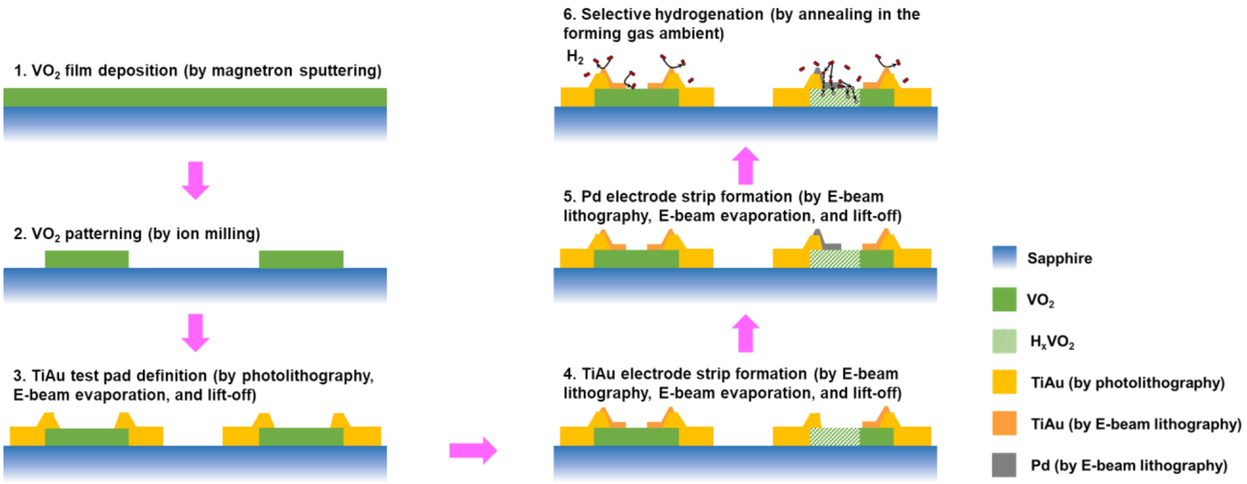

Fig. S29. Fabrication process flow of homotypic volatile and non-volatile (H_x)VO_2 components integrated on a single sapphire substrate. All devices were fabricated with a two-terminal geometry. With the combination of TiAu+TiAu and TiAu+Pd electrode pairs, the devices can exhibit volatile and non-volatile electronic behavior, respectively.

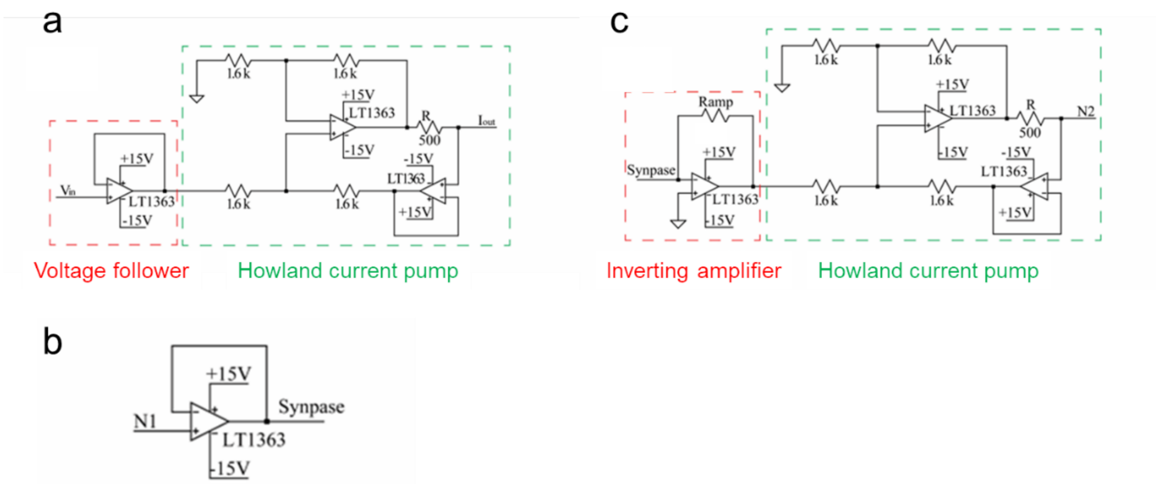

Fig. S30. Circuit components in the feedforward excitation and inhibition neural circuits. (a) Voltage-to-current converter connected between the pre-synaptic neuron and the arbitrary function generator. The voltage-to-current converter consists of three LT1363 operation amplifiers (op-amps). The first stage acts as a voltage follower to enforce an almost ideal voltage source since the arbitrary signal generator has an inherent output resistance of 50 Ω. The second stage is an improved Howland current pump. The output currents can be calculated with the equation: $I_{out} = V_{in}/R$, where R is 500 Ω. Therefore, the voltage-to-current conversion ratio of 1 V to 2 mA. (b) Isolator (Iso1) between the pre-synaptic neuron and the synapse. An LT1363 op-amp is used here as a voltage follower to avoid any loading effects when the signal is transferred between the pre-synaptic neuron and the synapse (c) Isolator (Iso2) between the synapse and the post-synaptic neuron. Three LT1363 op-amps are employed here. The first stage is an inverting amplifier. It amplifies the voltage drop across the synapse per the gain, the absolute value of which is defined as the synaptic weight ($w = R_{amp}/R_{synapse}$), where R_{amp} is the resistance across the op-amp, and $R_{synapse}$ is effective synapse resistance. The second stage is the same Howland current pump in the voltage-to-current converter for injecting current spikes to the post-synaptic neuron.

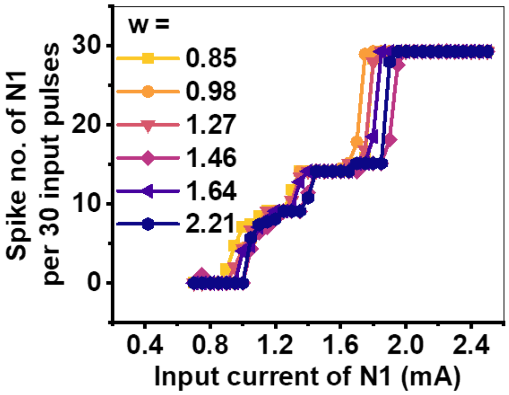

Fig. S31. Measured input-current-dependent firing probability of N1 under different synaptic weights. N1 fires more frequently with the rise of input pulse amplitude. Due to the employment of isolators, N1 is less affected by the synaptic weight in the circuit with a feedforward motif.

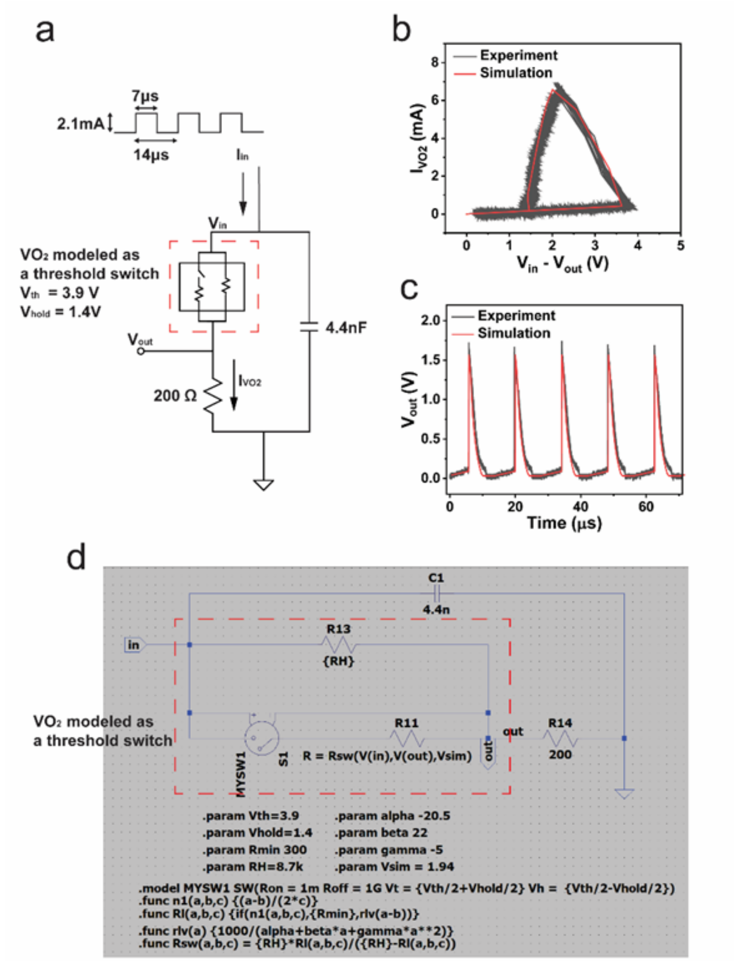

Fig. S32. Neuronal component modeling. (a) An equivalent circuit comprised of a threshold switch in series with a 200 Ω resistor and in parallel with a 4.4 nF capacitor to mimic the spiking behavior of neurons. (b) Comparison of simulated and experimental I-V characteristics. (c) Comparison of simulated and experimental spiking characteristics. The input current pulses have an A_p of 2.1 mA, a t_p of 7 μs, and a D_p of 50%. (d) LTspice simulation circuit and parameters for the single neuronal component.

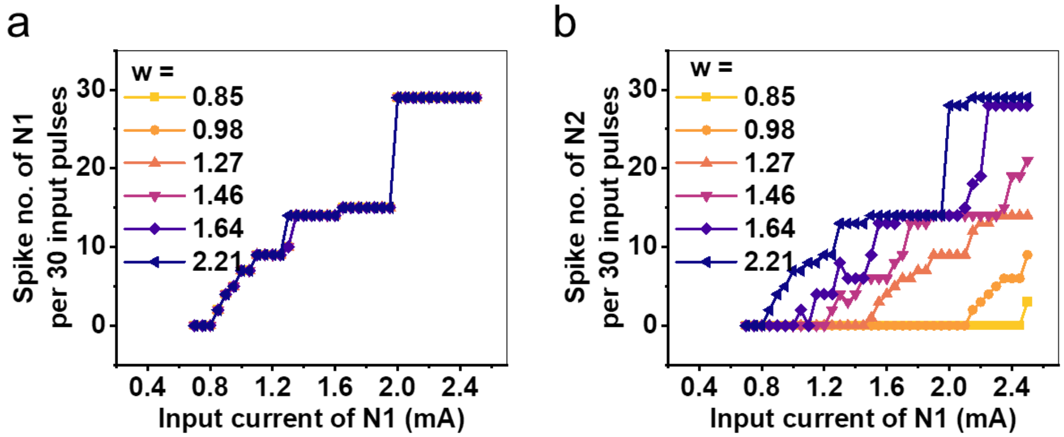

Fig. S33. SPICE simulation on the feedforward excitation/inhibition neural circuit with connected (H_x)VO_2 neurons and synapses on a single chip. Simulated input-current-dependent spike number of (a) N1 and (b) N2 per 30 input current pulses under different synaptic weights.

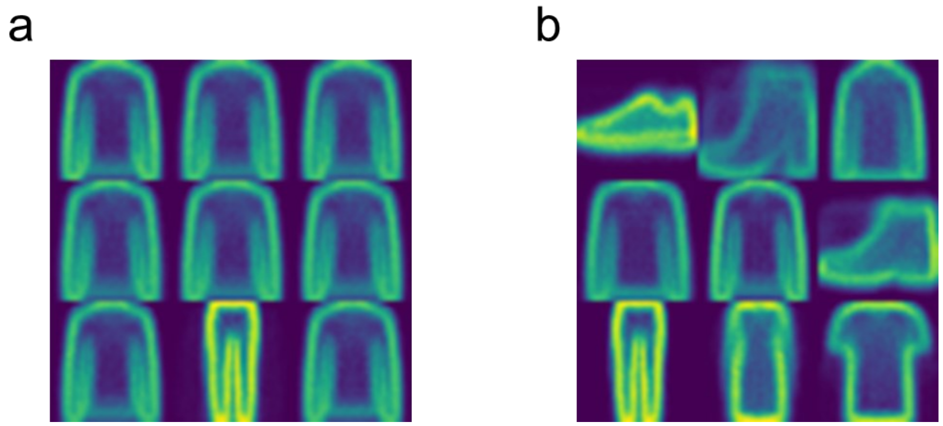

Fig. S34. Effect of the inhibitory connections on the network. (a) When there are no inhibitory connections, there is no competition among the neurons and most neurons learn the same pattern. This greatly reduces the generalization capability of the network. (b) In comparison, the presence of inhibitory connections induces competition among neurons to learn different patterns and thus, greater generalization over the dataset.

Table S1. LTspice simulation parameters for single neuronal component

Parameters	Values
V_{th} (V)	3.9
V_{hold} (V)	1.4
R_H (Ω)	8.7k
R_{min} (Ω)	300
V_{sim} (V)	1.94
α (Ω^{-1})	-20.5
β ($V^{-1} \times \Omega^{-1}$)	22
γ ($V^{-2} \times \Omega^{-1}$)	-5

Table S2. LTspice simulation parameters for the circuits

Parameters	N1 Values	N2 Values
V_{th} (V)	3.9	3.6
V_{hold} (V)	1.4	1.5
R_H (Ω)	8.7k	8.7k
R_{min} (Ω)	300	300
V_{sim} (V)	1.94	1.6
α (Ω^{-1})	-20.5	-20.5
β ($V^{-1} \times \Omega^{-1}$)	22	22
γ ($V^{-2} \times \Omega^{-1}$)	-5	-5

Table S3. Network-level simulation parameters

Parameters	Values
No. of excitatory neurons	1600 (MNIST)<br>400 (Fashion-MNIST)
Pre-synaptic learning rate, η_{pre}	10^{-4} (MNIST)<br>4×10^{-5} (Fashion-MNIST)
Post-synaptic learning rate, η_{post}	10^{-2} (MNIST)<br>4×10^{-3} (Fashion-MNIST)
Spike trace decay time constant, τ_{trace}	20ms
Quantization precision, N	4
No. of training epochs	10 (MNIST)<br>3 (Fashion-MNIST)

Supplementary Text

1. Proposed mechanisms regarding non-volatile switching behavior shown in Fig. 3(d)

In brief, the particular "8"-like I-V behavior in the positive polarity of Fig. 3(d) is determined by the partially hydrogenated VO_2 channel, where resistive switching and threshold switching co-exist and threshold switching takes place after the occurrence of resistive switching. The hypothesized mechanistic details are elaborated below.

In the as-hydrogenated device, the protons reside near the catalytic Pd electrode. The protons can rapidly drift under the external electric field and form conductive pathways between Pd and TiAu, leading to a sharp decrease in device resistance. During the non-volatile switching process, under a positive bias, the protons continue to drift towards TiAu (Stage 1 in Fig. 3(d)), and conducting pathways near the Pd electrode are ruptured. Then, pristine-like VO_2 dominates this region and the device is reset by switching from the LRS to the HRS (Stage 2). Once the sweeping voltage further increases to the V_{th} (~2.4 V in Fig. 3(d)) of the E-IMT in this pristine-like VO_2 region, the device starts to show threshold switching (Stage 3). In the backward sweep from 3 V to 0 V, the volatile threshold switching disappears when the voltage is below the V_{hold} (~0.9 V in Fig. 3(d)) for the E-IMT. However, the device is still biased in the positive polarity and so the pristine-like VO_2 region near Pd remains insulating and the device stays at HRS (Stage 4). Subsequently, the bias polarity becomes negative. The protons start to migrate from TiAu to Pd, and the pristine-like VO_2 region is gradually protonated (Stage 5). Once the proton filaments between Pd and TiAu are reconnected, the device shows a current jump and is set from the HRS to the LRS (Stage 6). Upon further negative sweep, the protons continue to drift towards Pd. Therefore, the device can remain at the LRS (Stage 7). The above stages describe the non-volatile switching behavior in the hydrogenated TiAu+Pd device.

2. Details of material characterizations

X-ray diffraction (XRD): XRD was performed at room temperature using an Empyrean Powder X-ray diffractometer from Malvern Panalytical with a Cu X-ray source (λ=1.5406 Å for Kα).

Raman spectroscopy: Raman spectra were measured at room temperature by a Renishaw in-Via Raman spectrometer equipped with a 532-nm laser.

Conducting atomic force microscopy (C-AFM): Surface conductivity was probed using an Asylum Research MFP-3D atomic force microscope under contact mode imaging.

Scanning transmission electron microscopy (STEM) and electron energy loss spectroscopy (EELS): Samples were prepared using the *in-situ* lift-out method in the Helios 600 DualBeam instrument operating at 30 keV to obtain electron-transparent samples. A final clean-up at 5 keV was performed to remove part of the amorphous material on both sides of the lamellas. Samples were observed in a double aberration corrected JEOL ARM 200F cold FEG microscope operating at 200 keV in STEM mode. A semi-convergence angle of 21 mrad and a current of 60 pA were used. The inner and outer detector angles were set to 67 mrad and 275 mrad, respectively (High Angle Angular Dark-Field mode), leading to the Z-contrast imaging conditions. The EELS experiments were performed using the GIF Continuum spectrometer and the K3 direct detection camera in counting mode. The energy resolution was estimated at around 0.75 eV. The EELS spectra were gently denoised using the Principal Component Analysis method (PCA) keeping the first 10 components.

3. Methodology for density functional calculations

Density functional calculations were conducted using Vienna *ab initio* Simulation Package (VASP) with projector augmented waves in the generalized gradient approximation using the Perdew-Burke-Ernzerhof exchange correlation function (*52*). The rotationally invariant form of Generalized Gradient Approximation (GGA) + U from Refs. (*24, 53*) with U = 4 eV and J = 0.6 eV was used to treat the strong Coulomb repulsion among the V 3d electrons for VO_2, where U is the on-site Coulomb parameter and J is the on-site exchange parameter. The plane wave cut-off energy was set to 600 eV in all the calculations. The Brillioun zone sampled at Γ point with a *k*-point density (*54*) was defined by $n_{\text{atoms}} \times n_{\text{kpoints}}$ of 1000 and 10000, for structural relaxation and density of states calculations, respectively. The electronic convergence criterion for the total energies was set to 10^{-6} eV. The pristine structures of monoclinic and rutile polymorphs were collected from OQMD database (*55*). For low concentration of H dopant simulation calculations, we considered a supercell of 96 atoms for both the monoclinic (2×2×2) and rutile (2×2×4) phases. For medium concentration of H dopant calculation, we used the primitive lattice of monoclinic cell and for rutile phase we used a simulation cell of 1×1×2. Before carrying out the calculations of the electronic properties of the H-doped monoclinic and rutile phases, we performed cell shape, volume, and the ionic relaxations using the conjugate gradient approximations (*56*) as implemented in VASP until the Hellmann-Feynmann forces become less than 0.01 eV/Å. Symmetry was turned off and a smearing of width 5 meV was used in all the DFT calculations. A relatively low values of mixing parameters (AMIX = 0.1 and BMIX=0.001) was used in order to accelerate the convergence of electronic states.

REFERENCES AND NOTES

1. B. Yan, X. Cao, H. Li, paper presented at the 55th Annual Design Automation Conference, San Francisco, CA, 24 June 2018.

2. Y. Wang, K.-M. Kang, M. Kim, H.-S. Lee, R. Waser, D. Wouters, R. Dittmann, J. J. Yang, H.-H. Park, Mott-transition-based RRAM. *Mater. Today* **28**, 63–80 (2019).

3. M. S. Nikoo, R. Soleimanzadeh, A. Krammer, G. M. Marega, Y. Park, J. Son, A. Schueler, A. Kis, P. J. Moll, E. Matioli, Electrical control of glass-like dynamics in vanadium dioxide for data storage and processing. *Nat. Electron.* **5**, 596–603 (2022).

4. J. Lin, S. Sonde, C. Chen, L. Stan, K. Achari, S. Ramanathan, S. Guha, paper presented at 2016 IEEE International Electron Devices Meeting (IEDM), San Francisco, CA, 3 to 7 December 2016.

5. P.-Y. Chen, J.-S. Seo, Y. Cao, S. Yu, paper presented at 2016 IEEE/ACM International Conference on Computer-Aided Design (ICCAD), Austin, TX, 7 to 10 November 2016.

6. M. Jerry, W.-Y. Tsai, B. Xie, X. Li, V. Narayanan, A. Raychowdhury, S. Datta, paper presented at 2016 74th Annual Device Research Conference (DRC), Newark, DE, 19 to 22 June 2016.

7. E. Corti, J. A. Cornejo Jimenez, K. M. Niang, J. Robertson, K. E. Moselund, B. Gotsmann, A. M. Ionescu, S. Karg, Coupled VO_2 oscillators circuit as analog first layer filter in convolutional neural networks. *Front. Neurosci.* **15**, 628254 (2021).

8. M. D. Pickett, G. Medeiros-Ribeiro, R. S. Williams, A scalable neuristor built with Mott memristors. *Nat. Mater.* **12**, 114–117 (2013).

9. S. Oh, Y. Shi, J. Del Valle, P. Salev, Y. Lu, Z. Huang, Y. Kalcheim, I. K. Schuller, D. Kuzum, Energy-efficient Mott activation neuron for full-hardware implementation of neural networks. *Nat. Nanotechnol.* **16**, 680–687 (2021).

10. J. Park, C. Oh, J. Son, Anisotropic ionic transport-controlled synaptic weight update by protonation in a VO_2 transistor. *J. Mater. Chem. C* **9**, 2521–2529 (2021).

11. G. Li, D. Xie, H. Zhong, Z. Zhang, X. Fu, Q. Zhou, Q. Li, H. Ni, J. Wang, E.-j. Guo, M. He, C. Wang, G. Yang, K. Jin, C. Ge, Photo-induced non-volatile VO_2 phase transition for neuromorphic ultraviolet sensors. *Nat. Commun.* **13**, 1729 (2022).

12. Y. J. Lee, K. Hong, K. Na, J. Yang, T. H. Lee, B. Kim, C. W. Bark, J. Y. Kim, S. H. Park, S. Lee, H. W. Jang, Nonvolatile control of metal-insulator transition in VO_2 by ferroelectric gating. *Adv. Mater.* **34**, 2203097 (2022).

13. R. Yuan, Q. Duan, P. J. Tiw, G. Li, Z. Xiao, Z. Jing, K. Yang, C. Liu, C. Ge, R. Huang, Y. Yang, A calibratable sensory neuron based on epitaxial VO_2 for spike-based neuromorphic multisensory system. *Nat. Commun.* **13**, 3973 (2022).

14. Z. Wang, S. Joshi, S. Savel'ev, W. Song, R. Midya, Y. Li, M. Rao, P. Yan, S. Asapu, Y. Zhuo, H. Jiang, P. Lin, C. Li, J. H. Yoon, N. K. Upadhyay, J. Zhang, M. Hu, J. P. Strachan, M. Barnell, Q. Wu, H. Wu, R. S. Williams, Q. Xia, J. J. Yang, Fully memristive neural networks for pattern classification with unsupervised learning. *Nat. Electron.* **1**, 137–145 (2018).

15. Q. Duan, Z. Jing, X. Zou, Y. Wang, K. Yang, T. Zhang, S. Wu, R. Huang, Y. Yang, Spiking neurons with spatiotemporal dynamics and gain modulation for monolithically integrated memristive neural networks. *Nat. Commun.* **11**, 3399 (2020).

16. J. Woo, P. Wang, S. Yu, Integrated crossbar array with resistive synapses and oscillation neurons. *IEEE Electron Device Lett.* **40**, 1313–1316 (2019).

17. Y. Gong, H. Yuan, C.-L. Wu, P. Tang, S.-Z. Yang, A. Yang, G. Li, B. Liu, J. van de Groep, M. L. Brongersma, M. F. Chisholm, S.-C. Zhang, W. Zhou, Y. Cui, Spatially controlled doping of two-dimensional SnS_2 through intercalation for electronics. *Nat. Nanotechnol.* **13**, 294–299 (2018).

18. H.-P. Komsa, J. Kotakoski, S. Kurasch, O. Lehtinen, U. Kaiser, A. V. Krasheninnikov, Two-dimensional transition metal dichalcogenides under electron irradiation: Defect production and doping. *Phys. Rev. Lett.* **109**, 035503 (2012).

19. H. Fang, M. Tosun, G. Seol, T. C. Chang, K. Takei, J. Guo, A. Javey, Degenerate n-doping of few-layer transition metal dichalcogenides by potassium. *Nano Lett.* **13**, 1991–1995 (2013).

20. C. Ahn, A. Bhattacharya, M. Di Ventra, J. N. Eckstein, C. D. Frisbie, M. Gershenson, A. Goldman, I. Inoue, J. Mannhart, A. J. Millis, A. F. Morpurgo, D. Natelson, J.-M. Triscone, Electrostatic modification of novel materials. *Rev. Mod. Phys.* **78**, 1185–1212 (2006).

21. P. A. Lee, N. Nagaosa, X.-G. Wen, Doping a Mott insulator: Physics of high-temperature superconductivity. *Rev. Mod. Phys.* **78**, 17–85 (2006).

22. Y. Zhou, X. Guan, H. Zhou, K. Ramadoss, S. Adam, H. Liu, S. Lee, J. Shi, M. Tsuchiya, D. D. Fong, S. Ramanathan, Strongly correlated perovskite fuel cells. *Nature* **534**, 231–234 (2016).

23. H. Yoon, M. Choi, T.-W. Lim, H. Kwon, K. Ihm, J. K. Kim, S.-Y. Choi, J. Son, Reversible phase modulation and hydrogen storage in multivalent VO_2 epitaxial thin films. *Nat. Mater.* **15**, 1113–1119 (2016).

24. J. Wei, H. Ji, W. Guo, A. H. Nevidomskyy, D. Natelson, Hydrogen stabilization of metallic vanadium dioxide in single-crystal nanobeams. *Nat. Nanotechnol.* **7**, 357–362 (2012).

25. V. Andreev, V. Kapralova, V. Klimov, Effect of hydrogenation on the metal-semiconductor phase transition in vanadium dioxide thin films. *Phys. Solid State* **49**, 2318–2322 (2007).

26. Y. LeCun, C. C. Christopher, J.C. Burges, The MNIST database of handwritten digits (2010); http://yann.lecun.com/exdb/mnist/.

27. H. Xiao, K. Rasul, R. Vollgraf, Fashion-MNIST: a Novel Image Dataset for Benchmarking Machine Learning Algorithms (2017); https://arxiv.org/abs/1708.07747.

28. J. Chenavas, J. Joubert, J. Capponi, M. Marezio, Synthese de nouvelles phases denses d'oxyhydroxydes M^{3+} OOH des metaux de la premiere serie de transition, en milieu hydrothermal à tres haute pression. *J. Solid State Chem.* **6**, 1–15 (1973).

29. Y. Chen, Z. Wang, S. Chen, H. Ren, L. Wang, G. Zhang, Y. Lu, J. Jiang, C. Zou, Y. Luo, Non-catalytic hydrogenation of VO_2 in acid solution. *Nat. Commun.* **9**, 818 (2018).

30. P. Sermon, G. Bond, Hydrogen spillover. *Catal. Rev.* **8**, 211–239 (1974).

31. T. S. Kasırga, J. M. Coy, J. H. Park, D. H. Cobden, Visualization of one-dimensional diffusion and spontaneous segregation of hydrogen in single crystals of VO_2. *Nanotechnology* **27**, 345708 (2016).

32. X. Duan, S. T. White, Y. Cui, F. Neubrech, Y. Gao, R. F. Haglund, N. Liu, Reconfigurable multistate optical systems enabled by VO_2 phase transitions. *ACS Photonics* **7**, 2958–2965 (2020).

33. S. Chen, Z. Wang, L. Fan, Y. Chen, H. Ren, H. Ji, D. Natelson, Y. Huang, J. Jiang, C. Zou, Sequential insulator-metal-insulator phase transitions of VO_2 triggered by hydrogen doping. *Phys. Rev. B* **96**, 125130 (2017).

34. D. Delgado, G. Hefter, M. Minakshi, Hydrogen Generation, in *Alternative Energies* (Springer, 2013), pp. 141–161.

35. P. Schilbe, Raman scattering in VO_2. *Physica B Condens. Matter.* **316–317**, 600–602 (2002).

36. S. Zhang, J. Y. Chou, L. J. Lauhon, Direct correlation of structural domain formation with the metal insulator transition in a VO2 nanobeam. *Nano Lett.* **9**, 4527–4532 (2009).

37. P.-Y. Chen, B. Lin, I.-T. Wang, T.-H. Hou, J. Ye, S. Vrudhula, J.-S. Seo, Y. Cao, S. Yu, paper presented at 2015 IEEE/ACM International Conference on Computer-Aided Design (ICCAD), Austin, TX, 2 to 6 November 2015.

38. F.-X. Liang, I.-T. Wang, T.-H. Hou, Progress and benchmark of spiking neuron devices and circuits. *Adv. Intell. Syst.* **3**, 2100007 (2021).

39. I. M. Pandiev, Analysis and design of voltage-controlled current sources for a grounded load. *Int. J. Circuit Theory Appl.* **43**, 756–775 (2015).

40. A. Mahnam, H. Yazdanian, M. M. Samani, Comprehensive study of Howland circuit with non-ideal components to design high performance current pumps. *Measurement* **82**, 94–104 (2016).

41. Y. Fu, Y. Zhou, X. Huang, B. Dong, F. Zhuge, Y. Li, Y. He, Y. Chai, X. Miao, Reconfigurable synaptic and neuronal functions in a V/VOx/HfWOx/Pt memristor for nonpolar spiking convolutional neural network. *Adv. Funct. Mater.* **32**, 2111996 (2022).

42. G.-Q. Bi, M.-m. Poo, Synaptic modifications in cultured hippocampal neurons: Dependence on spike timing, synaptic strength, and postsynaptic cell type. *J. Neurosci.* **18**, 10464–10472 (1998).

43. P. U. Diehl, M. Cook, Unsupervised learning of digit recognition using spike-timing-dependent plasticity. *Front. Comput. Neurosci.* **9**, 99 (2015).

44. Q. Zhu, Z. Wang, An image clustering auto-encoder based on predefined evenly-distributed class centroids and MMD distance. *Neural Process Lett.* **51**, 1973–1988 (2020).

45. P. Ferré, F. Mamalet, S. J. Thorpe, Unsupervised feature learning with winner-takes-all based stdp. *Front. Comput. Neurosci.* **12**, 24 (2018).

46. S. R. Kheradpisheh, M. Ganjtabesh, S. J. Thorpe, T. Masquelier, STDP-based spiking deep convolutional neural networks for object recognition. *Neural Netw.* **99**, 56–67 (2018).

47. H. Hazan, D. J. Saunders, H. Khan, D. Patel, D. T. Sanghavi, H. T. Siegelmann, R. Kozma, BindsNET: A machine learning-oriented spiking neural networks library in python. *Front. Neurosci.* **12**, 89 (2018).

48. A. Morrison, M. Diesmann, W. Gerstner, Phenomenological models of synaptic plasticity based on spike timing. *Biol. Cybern.* **98**, 459–478 (2008).

49. S. Hu, G. Qiao, T. Chen, Q. Yu, Y. Liu, L. Rong, Quantized STDP-based online-learning spiking neural network. *Neural Comput. Appl.* **33**, 12317–12332 (2021).

50. S. Chen, Z. Wang, H. Ren, Y. Chen, W. Yan, C. Wang, B. Li, J. Jiang, C. Zou, Gate-controlled VO_2 phase transition for high-performance smart windows. *Sci. Adv.* **5**, eaav6815 (2019).

51. W. Yi, K. K. Tsang, S. K. Lam, X. Bai, J. A. Crowell, E. A. Flores, Biological plausibility and stochasticity in scalable VO_2 active memristor neurons. *Nat. Commun.* **9**, 4661 (2018).

52. J. P. Perdew, K. Burke, M. Ernzerhof, Generalized gradient approximation made simple. *Phys. Rev. Lett.* **77**, 3865 (1996), 3868.

53. Y. Cui, S. Shi, L. Chen, H. Luo, Y. Gao, Hydrogen-doping induced reduction in the phase transition temperature of VO_2: A first-principles study. *Phys. Chem. Chem. Phys.* **17**, 20998–21004 (2015).

54. S. Manna, P. Gorai, G. L. Brennecka, C. V. Ciobanu, V. Stevanović, Large piezoelectric response of van der Waals layered solids. *J. Mater. Chem. C* **6**, 11035–11044 (2018).

55. J. E. Saal, S. Kirklin, M. Aykol, B. Meredig, C. Wolverton, Materials design and discovery with high-throughput density functional theory: The open quantum materials database (OQMD). *Jom* **65**, 1501–1509 (2013).

56. I. Štich, R. Car, M. Parrinello, S. Baroni, Conjugate gradient minimization of the energy functional: A new method for electronic structure calculation. *Phys. Rev. B* **39**, 4997 (1989), 5004.
